# Universal digital high-resolution melting for the detection of pulmonary mold infections

**DOI:** 10.1128/jcm.01476-23

**Published:** 2024-05-02

**Authors:** Tyler Goshia, April Aralar, Nathan Wiederhold, Jeffrey D. Jenks, Sanjay R. Mehta, Aprajita Karmakar, Monish E.S., Ankit Sharma, Haoxiang Sun, Refilwe Kebadireng, P. Lewis White, Mridu Sinha, Martin Hoenigl, Stephanie I. Fraley

**Affiliations:** 1 Department of Bioengineering, University of California San Diego, San Diego, California, USA; 2 Department of Pathology, University of Texas Health Science Center, San Antonio, Texas, USA; 3 Department of Medicine, Duke University School of Medicine, Durham, North Carolina, USA; 4 Durham County Department of Public Health, Durham, North Carolina, USA; 5 Department of Medicine, University of California San Diego, San Diego, California, USA; 6 San Diego Veterans Administration Medical Center, San Diego, California, USA; 7 MelioLabs Inc., Santa Clara, California, USA; 8 Public Health Wales Microbiology Cardiff, Cardiff University, UHW, Cardiff, United Kingdom; 9 Centre for Trials Research, Division of Infection and Immunity, Cardiff University, UHW, Cardiff, United Kingdom; 10 Department of Internal Medicine, Medical University of Graz, Graz, Austria; 11 ECMM Excellence Center for Medical Mycology, Medical University of Graz, Graz, Austria; University of Utah, Salt Lake City, Utah, USA

**Keywords:** IMI, dPCR, HRM, machine learning

## Abstract

Invasive mold infections (IMIs) are associated with high morbidity, particularly in immunocompromised patients, with mortality rates between 40% and 80%. Early initiation of appropriate antifungal therapy can substantially improve outcomes, yet early diagnosis remains difficult to establish and often requires multidisciplinary teams evaluating clinical and radiological findings plus supportive mycological findings. Universal digital high-resolution melting (U-dHRM) analysis may enable rapid and robust diagnoses of IMI. A universal fungal assay was developed for U-dHRM and used to generate a database of melt curve signatures for 19 clinically relevant fungal pathogens. A machine learning algorithm (ML) was trained to automatically classify these pathogen curves and detect novel melt curves. Performance was assessed on 73 clinical bronchoalveolar lavage samples from patients suspected of IMI. Novel curves were identified by micropipetting U-dHRM reactions and Sanger sequencing amplicons. U-dHRM achieved 97% overall fungal organism identification accuracy and a turnaround time of ~4 hrs. U-dHRM detected pathogenic molds (*Aspergillus*, *Mucorales*, *Lomentospora*, and *Fusarium*) in 73% of 30 samples classified as IMI, including mixed infections. Specificity was optimized by requiring the number of pathogenic mold curves detected in a sample to be *>*8 and a sample volume to be 1 mL, which resulted in 100% specificity in 21 at-risk patients without IMI. U-dHRM showed promise as a separate or combination diagnostic approach to standard mycological tests. U-dHRM’s speed, ability to simultaneously identify and quantify clinically relevant mold pathogens in polymicrobial samples, and detect emerging opportunistic pathogens may aid treatment decisions, improving patient outcomes.

**IMPORTANCE:**

Improvements in diagnostics for invasive mold infections are urgently needed. This work presents a new molecular detection approach that addresses technical and workflow challenges to provide fast pathogen detection, identification, and quantification that could inform treatment to improve patient outcomes.

## INTRODUCTION

Invasive mold infections (IMI) cause millions of infections globally and account for an estimated 1.6 million deaths annually (1). Patients at risk from IMIs, including both severely immunocompromised and also more immunocompetent individuals (2), are increasing. IMIs in more immunocompetent persons/those receiving systemic corticosteroids are characterized by early tissue invasive growth in the lungs with bloodstream invasion potentially occurring later although not universally, while early angioinvasive growth is more common in severely immunocompromised persons (3). Ground truth IMIs have characteristically been very difficult to diagnose before death, with rates of pre-mortem diagnosis ranging from 12% to 60% (4). Histopathologic examination and culture of tissue or bronchoalveolar lavage fluid (BALF) are considered the reference standard for IMI diagnosis but are slow, with histopathology often available only at autopsy, while culture has poor sensitivity (5). Incubation of fungal cultures for 4 weeks is considered best practice to maximize the recovery of slow growing species, with most detected by day 14 (6). BALF antigen tests, such as galactomannan (GM), can be helpful but are only positive for a limited number of specific mold organisms and are further limited by variable turnaround times (TAT) and lower sensitivity for individuals on mold-active antifungal prophylaxis or treatment (2). PCR assays are currently advancing as recommended complementary diagnostic tools due to their high sensitivity and specificity, ability to identify mutations associated with antifungal resistance, and ability to detect non-Aspergillus mold infections. The T2 Candida Panel (T2 Biosystems, Lexington, MA, USA), BioFire FilmArray Meningitis/Encephalitis (ME) Panel, and BioFire FilmArray Blood Culture Identification (BCID) Panel (BioFire Diagnostics, Salt Lake City, Utah, USA) are FDA-approved commercially available assays for whole blood, positive blood culture, and/or cerebrospinal fluid that have demonstrated excellent performance with swift turnaround times of 1–4 hours but only detect a limited panel of yeast pathogens (2). Pan-fungal assays and assays capable of detecting rare or novel fungi are limited to next generation sequencing (NGS)-based approaches, which suffer from high complexity that results in a send-out format and long turnaround times (2). Also, a recent study that applied both targeted NGS and metagenomic NGS to BALF samples found that both approaches failed to identify true fungal-positive cases (7). The absence of rapid and accessible fungal diagnostics often results in empiric utilization of systemic antifungals, mostly targeted against *Aspergillus* spp., some of which are lacking activity against other molds (8). As a prominent example, mucormycosis diagnosis is particularly challenging (9). Pulmonary mucormycosis remains one of the most common non-*Aspergillus* mold infections in many US centers and has been globally and particularly in India on the rise as a complication in COVID-19 patients (10). There is hope on the horizon with *Mucorales* PCR now starting to be implemented in some clinical centers (11). However, currently, IMIs are often diagnosed and treated too late, leading to high mortality rates of 40%–80%. It is estimated that 80% of patients could be saved with rapid diagnostics to inform early and targeted treatment (12).

**Fig 1:**
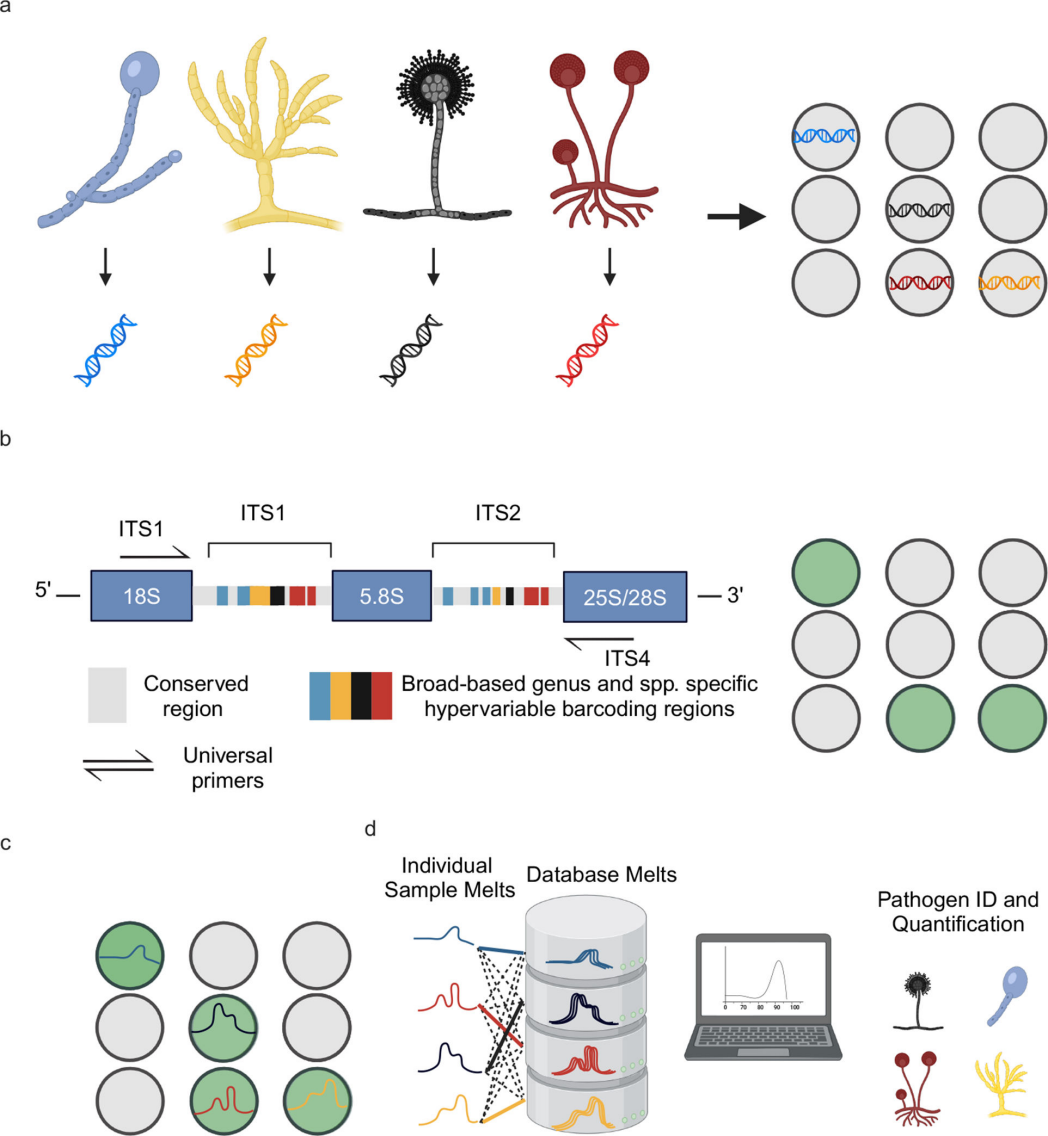
U-dHRM technology overview. (**a**) Extraction of genomic DNA and digital loading. (**b**) Universal amplification of fungal internal transcribed spacer (ITS) barcoding region leading to a fluorescence increase in each positive reaction well. (**c**) Barcode sequence-defined melt curve signatures. (**d**) Automatic identification of each known pathogen melt curve and detection of novel melt curves using machine learning. Created with BioRender.com.

Universal digital high-resolution melting (U-dHRM) to detect mold pathogens in BALF may be a promising probe-free diagnostic approach applicable without *a priori* knowledge of anticipated fungal organisms that could serve as a powerful complementary diagnostic tool upstream of sequencing to achieve rapid and near point-of-care diagnosis to inform treatment decisions and improve patient outcomes. This approach consists of a single closed-tube test that integrates universal amplification of pathogen barcoding sequences in a digital polymerase chain reaction (dPCR) format with high-resolution melting (HRM) of DNA and machine learning (Fig. 1) (13–16). Unlike NGS approaches, U-dHRM eliminates the need for post processing, thereby preventing external nucleic acid contamination and simplifying requirements for test operators. The integration and advancement of these techniques promise a unique combination of advantages: speed and breadth of detection, sensitivity and absolute quantification, and pathogen identification in polymicrobial samples (17, 18).

Here, we advanced the U-dHRM assay and database for the detection of IMI pathogens, advanced the machine learning algorithm to recognize database organism curves and also flag novel organism melt curves, and developed a dPCR reaction recovery method to Sanger sequence novel melt curves and expand the pathogen panel. We applied these advancements to test 75 clinical BALF samples, assessing the utility of this approach for IMI diagnosis compared with gold standard tests.

## MATERIALS AND METHODS

### ITS-Asp dPCR

ITS1 (5′-TCCGTAGGTGAACCTGCGG-3′) and ITS4 (5′-TCCTCCGCTTATTGATATGC-3′) universal primers multiplexed with Asp 1 (5′-CGGCCCTTAAATAGCCCGGTC-3′) and Asp 2 (5′-ACCCCCCTGAGCCAGTCCG-3′) were used to amplify the ITS universal region for all fungi and an *Aspergillus* specific region of the 18S gene. Each curve in dPCR originated from an individual partition containing single genomes or genome fragments. At least three dPCR chips were run for each organism type. ITS-Asp PCR was amplified using the following protocol: each 15 µL reaction mixture contained 0.1 µM of each primer (IDT, Coralville, IA), 0.2 mM deoxynucleoside triphosphate (dNTP) (Invitrogen, Carlsbad, CA), 1× Phusion GC PCR buffer (Thermo Scientific, Waltham, MA), 2.5× EvaGreen (Biotium, Fremont, CA), 0.02 U/µL Phusion polymerase (New England Biolabs, Ipswich, MA), ultrapure water (Quality Biological, Gaithersburg, MD), and 3 µL of genomic DNA from a final elution volume of 100 µL using the MolYsis Complete 5 kit (Molzym, Bremen, Germany). Thermocycling for quantitative PCR (qPCR)/dPCR and subsequent melt analysis were performed on a QuantStudio 3D real-time PCR system and a ProFlex 2× Flat Block Thermal Cycler (Applied Biosystems, Waltham, MA) using the QuantStudio 3D Digital PCR Chip (Applied Biosystems, Foster City, CA). The cycling conditions were as follows: hold at 98°C for 30 s, followed by 75 cycles of 98°C for 10 s, 61°C for 30 s, and 72°C for 60 s to ensure full endpoint amplification from single molecules (16, 19). At the end of cycling, there was a final extension step at 72°C for 5 min, which resulted in a total run time of approximately 3 hours of dPCR cycling. PCR amplification was followed by a melt cycle of an initial denaturation at 95°C for 15 s and then heating from 65°C to 95°C at a ramp rate of 0.2°C/s (15, 20).

### Control human β-actin PCR

Human beta actin primers, forward (5′-CGGCCTTGGAGTGTGTATTAAGTA-3′) and reverse (5′-TGCAAAGAACACGGCTAAGTGT-3′) were used to amplify the human β-actin gene. Each PCR was conducted in triplicate using the following protocol: each 15 µL reaction mixture contained 0.1 µM each primers (IDT, Coralville, IA), 0.2 mM dNTP (Invitrogen, Carlsbad, CA), 1× Phusion GC PCR buffer (Thermo Scientific, Waltham, MA), 2.5× EvaGreen (Biotium, Fremont, CA), 0.02 U/µL Phusion polymerase (New England Biolabs, Ipswich, MA), ultrapure water (Quality Biological, Gaithersburg, MD), and 3 µL of direct BALF sample liquid. Thermocycling for qPCR and subsequent melt analysis were performed on a QuantStudio 3D real-time PCR system (Applied Biosystems, Waltham, MA). The cycling conditions were as follows: hold at 98°C for 30 s, followed by 55 cycles of 98°C for 10 s, 66°C for 30 s, and 72°C for 45 s. At the end of cycling, there was a final extension step at 72° for 5 min. PCR amplification was followed by a melt cycle of an initial denaturation at 95°C for 15 s and then heating from 65°C to 95°C.

### DNA isolation for melt curve database generation

The following fungal strains were provided as clinical isolates by Dr. Nathan Weiderhold at the Department of Pathology University of Texas Health Science Center, San Antonio, TX: *Aspergillus terreus*, *Aspergillus nidulans*, *Aspergillus versicolor*, *Mucor circinelloides*, *Mucor velutinosus*, *Mucor plumbeus*, *Rhizopus arrhizus var. delemar*, *Rhizopus microsporus*, *Lomentospora prolificans*, *Scedosporium apiospermum*, *Scopulariopsis brevicaulis*, *Scopulariopsis candida*, and *Scopulariopsis gossypii. Aspergillus fumigatus*, *Aspergillus flavus*, *Aspergillus niger*, *Fusarium oxysporum*, *Cryptococcus neoformans*, *Candida krusei*, *Candida glabrata*, and *Candida albicans* were provided as clinical isolates from Dr. Sanjay Mehta at the San Diego VA Clinical Microbiology Laboratory. *Candida auris* was provided as a clinical isolate by Dr. Sharon Reed at the UCSD Center for Advanced Laboratory Medicine. For database generation, DNA was extracted using the Lucigen MasterPure Yeast DNA Purification Kit (Lucigen, Middleton, WI, USA). DNA concentration was measured by bio-spectrophotometer absorbance readings and diluted to the target concentrations.

### DNA isolation from clinical BALF samples

Prior to DNA isolation, direct PCR β-actin was run to assess lavage quality as described above. Clinical BALF sample DNA was isolated in approximately less than 1 hour using MolYsis Complete 5 Small Size Sample DNA Isolation (≤1 mL liquid) protocol (Molzym, Bremen, Germany). Each BALF sample was run in U-dHRM with Asp-ITS PCR conditions as described above.

### Control pig BALF and analytical validation

For control and analytical spike-in experiments, pig BALF was collected from euthanized pigs previously treated with antibiotics and anesthetized with ketamine/xylene/atropine. Ambu aScope 4 Broncho single-use bronchoscopes (Ambu A/S, Ballerup, Denmark) were used with 50 mL sterile isotonic irrigation 0.9% saline (NDC 0990-6138-22) for lavages. Pig BALF was used because healthy human BALF is not readily attainable. As the BALF collection procedure is invasive, it is not typically collected from healthy humans, and there are no synthetic or standardized BALF matrices for diagnostic development purposes. Prior to analytical validation, pig BALF was screened to be negative for target organisms by U-dHRM. Target organism spores were counted by a hemocytometer and plated to determine CFUs, and six 10-fold serial dilutions were conducted to achieve concentrations down to 1 CFU/mL, with concurrent no spike controls. *A. fumigatus* and *C. albicans* spores from each concentration were spiked into 2 mL of pig BALF to achieve the final concentrations of 10k, 1k, 100, 10, 1, and 1 spores (CFU)/mL of BALF.

### DNA sequencing

PCR products were prepared using ExoSAP-IT (Applied Biosystems, Foster City, CA) according to the manufacturer’s protocol and then sent for Sanger sequencing (GENEWIZ, San Diego, USA) using the same respective Asp and ITS forward primers described above.

### Image processing and data analysis

A sequence of raw fluorescence images was captured during the heating and melting procedure for each chip. Subsequently, these images underwent a sequence of image processing steps to identify and extract the individual wells within them along with their corresponding average intensity values. This 5–10-min procedure resulted in the translation of the average intensity measurements for each well across the entire set of images into a chronological array of values, thus creating a time series representation.

The original fluorescence time series, recognized as melt curves, underwent a twofold transformation: initially, they were converted into their respective derivatives, after which they were subjected to a smoothing process using a Savitzky-Golay filter. Furthermore, these smoothed derivative time series were classified as “Positive” if they exhibited a peak or local maxima beyond a temperature threshold of 85°C and with a minimum negative derivative of fluorescence over temperature (−dF/dT) value of 4. In this context, a “Positive” melt curve designates an instance where the presence of a particular fungal target is anticipated, whereas the remaining instances are categorized as “Negatives.”

Leveraging these identified “Positive” melt curves, a data set for machine learning purposes was constructed. Each time series within this data set represented a derivative melt curve that was smoothed using a Savitzky-Golay filter with the following parameters: a window length of 9 and a polynomial order of 3. These time series were then normalized using area under the curve normalization.

### Machine learning

**Fig 2:**
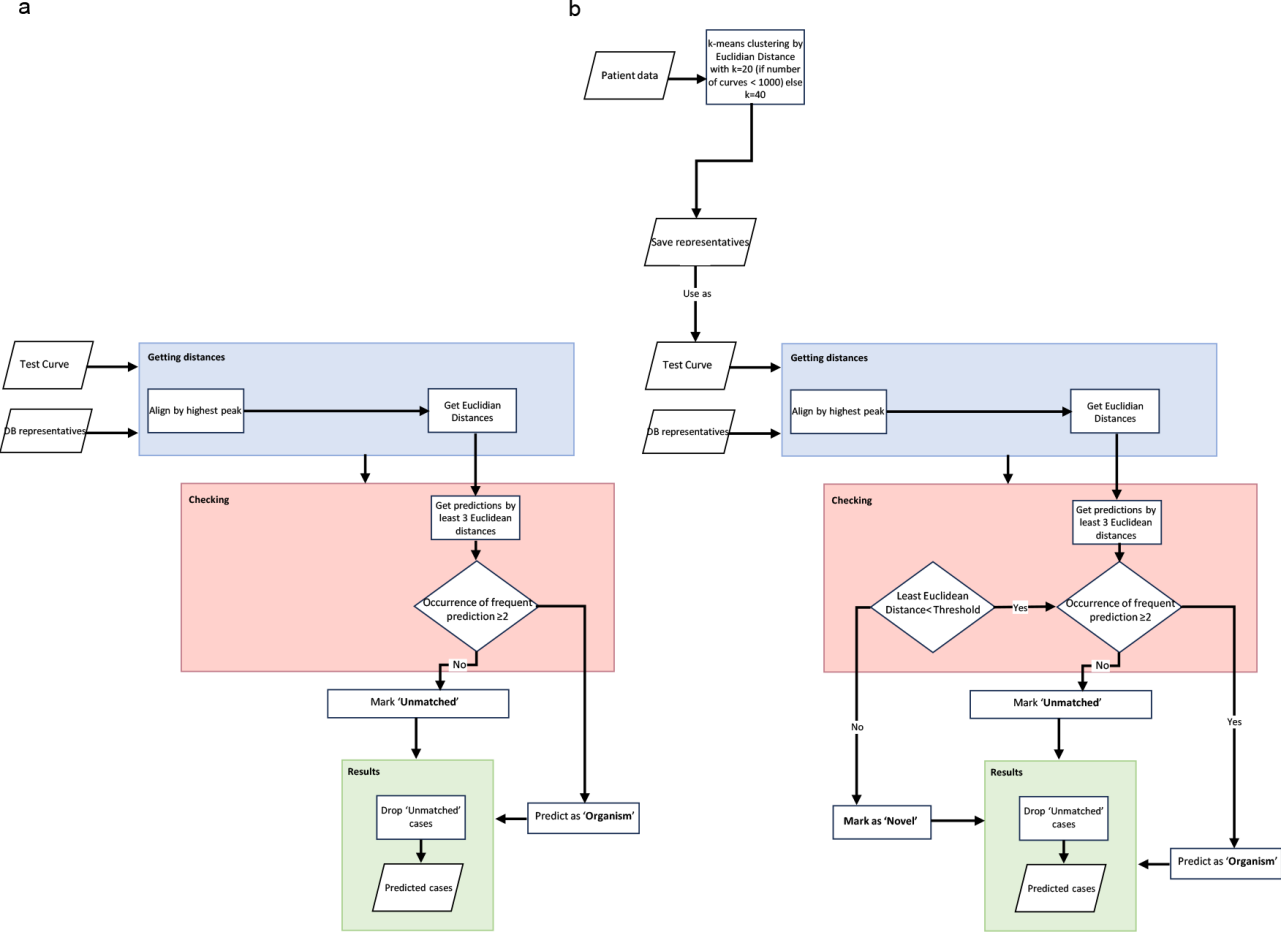
Machine learning process. (a) Flowchart for database sample testing. (b) Flowchart of patient sample testing differentiating between database classification and novelty detection.

We constructed a model based on our established database of organisms. This approach consists of a two-step procedure. The data set we employed comprises a comprehensive set of 10,000 melt curves attributed to each distinct organism. Within this data set, a subset of 10%, equating to 1,000 random melt curves, was selected and subjected to a time series Dynamic Time Warping (DTW) distance-based K-means clustering process, yielding a culmination of up to 50 representatives (21–23). Clusters housing fewer than 10 melt curves were excluded from consideration due to their susceptibility to noise-related interference. Owing to the substantial variability and inherent noise within the melt curves, we employed the K-means clustering technique as the initial step to identify pivotal clusters of variation, thereby yielding corresponding cluster centers that serve as robust and condensed representations of signals. These cluster centers are referred to as “DB representatives” in the flow charts in Fig. 2. The subsequent classification considers each cluster separately.

Another point to note is that instead of using the usual Euclidean distance-based K-means, we use DTW for both the cluster assignment and the averaging step of K-means. Temporal distortions (or shift) along the temperature (or time) axis causing well-to-well as well as chip-to-chip variations in melt curves are something inherent in HRM (13, 15, 24) and can be dealt with by using the various elastic distance measures for time series—among which the most popular one is DTW (13, 25) and its variations (26, 27). More specifically, as we use DTW distance, we employ a more suitable DTW-based Barycenter Averaging technique, as proposed by Petitjean et al. (23), for the K-means averaging step.

Subsequently, the second phase (Fig. 2) entailed the development of a classifier grounded in a 3 nearest neighbor (3NN) framework, leveraging the Euclidean distance as the defining metric. In this step, each test curve underwent alignment with every representative curve curated from the database (see blue boxes in Fig. 2a and b). Consequently, the KNN model was executed to discern the three nearest neighbors for each aligned test curve (see pink boxes in Fig. 2a and b) (28). The alignment procedure was deemed necessary to account for the potential shift-based discrepancies present among melt curves.

The outcome of this model provides predictions wherein concordance among the majority of neighbors designates a high-confidence classification. Conversely, instances in which all three nearest neighbors correspond to dissimilar organisms are categorized as low-confidence and consequently disregarded. Low-confidence instances can originate from either noisy signals or from novel curves that remain unrepresented within the existing database. The performance of classification was quantified through the assessment of accuracy for each organism.

Although in the literature, the terms *novelty detection* (ND), *anomaly detection* (AD), and *outlier detection* (OD) have been used interchangeably; AD and OD usually refer to noisy or erroneous signals while ND usually refers to a positive learning opportunity. That is, the novel point is treated as a resource for potential future use (29–31). Currently AD, ND, and OD are being studied under the common framework of Generalized Out of Distribution Detection (OOD) (32). Specifically for time series data, there is a significant amount of literature on AD but this research primarily focuses on finding point or subsequence anomalies within a large time series (33). As we have a larger number of smaller length time series, we consider each time series (melt curve) as a separate data point. We then use a distance-based OOD methodology for novelty detection [see section 5.3 of reference (32)] where the test curve is checked if it is outside of a certain standard deviations (threshold) away from each of the nearest three DB representatives (class cluster centers) obtained via 3NN step described above. If this check is successful, then the test point is certified as out of distribution and labeled as “novel.”

Furthermore, when dealing with patient samples, their time series were initially clustered utilizing the Euclidean-based K-means method (Fig. 2B, top left). The resultant cluster centers were then subjected to classification leveraging the pre-constructed 3NN-based classifier designed for the database curves.

#### Patients and samples

In this retrospective case control study, banked BALF samples originated from patients with various underlying diseases and clinical suspicion of invasive pulmonary aspergillosis (IPA) or IMI and GM and *Aspergillus* spp. culture testing ordered between 2015 and 2019 at the University of California San Diego (UCSD). IMI was classified according to the revised European Organization for Research and Treatment of Cancer (EORTC)/Mycoses Study Group (MSG) criteria (34) and slightly modified AspICU criteria (35) [i.e., including positive BALF fluid GM of 1.0 optical density index (ODI) as entry criterion (36, 37)] for patients in the intensive care unit (ICU) who did not fulfill EORTC/MSG host criteria. GM testing with the Platelia enzyme-linked immunosorbent assay (Bio-Rad Laboratories, Marnes-la-Coquette, France) was routinely and prospectively performed in all BALF samples before samples were stored at −70°C for up to 8 years. Based on classification, we retrospectively tested 75 patient BALF samples: 30 from patients diagnosed with proven (*n* = 1), probable (*n* = 25), or putative (*n* = 4) IPA infections and 45 from patients diagnosed as limited evidence or as not having IPA (*n* = 10 not classifiable, *n* = 4 possible IPA, and *n* = 31 classified as no IPA). Not-classifiable samples tested positive for mycological evidence and came from patients with clinical suspicion of IMI who did, however, not fulfill host factor criteria and/or did not present with typical radiological signs and were not admitted in the ICU. Direct β-actin PCR was used to access lavage quality according to previously published methods (38–40). Two samples (*n* = 1 possible and *n* = 1 no IPA) were excluded due to no human DNA being detected.

### Novelty detection and micromanipulator interrogation

Novel curves that were unrepresented within the existing database were identified with ML as described above. These curves’ physical X-Y on-chip coordinates were then identified using Melio Melt Inspector software (MelioLabs Inc., Santa Clara, CA, USA). A custom micromanipulator setup then sampled the target amplicons from individual or clusters of wells using a glass capillary. Sampled amplicons were either reamplified with Asp-ITS primers or sent directly for Sanger sequencing. Reamplified Asp-ITS dPCR chips were used to demonstrate the process of adding novel organisms to the established database.

## RESULTS

### Fungal U-dHRM assay development and analytical validation

**Fig 3:**
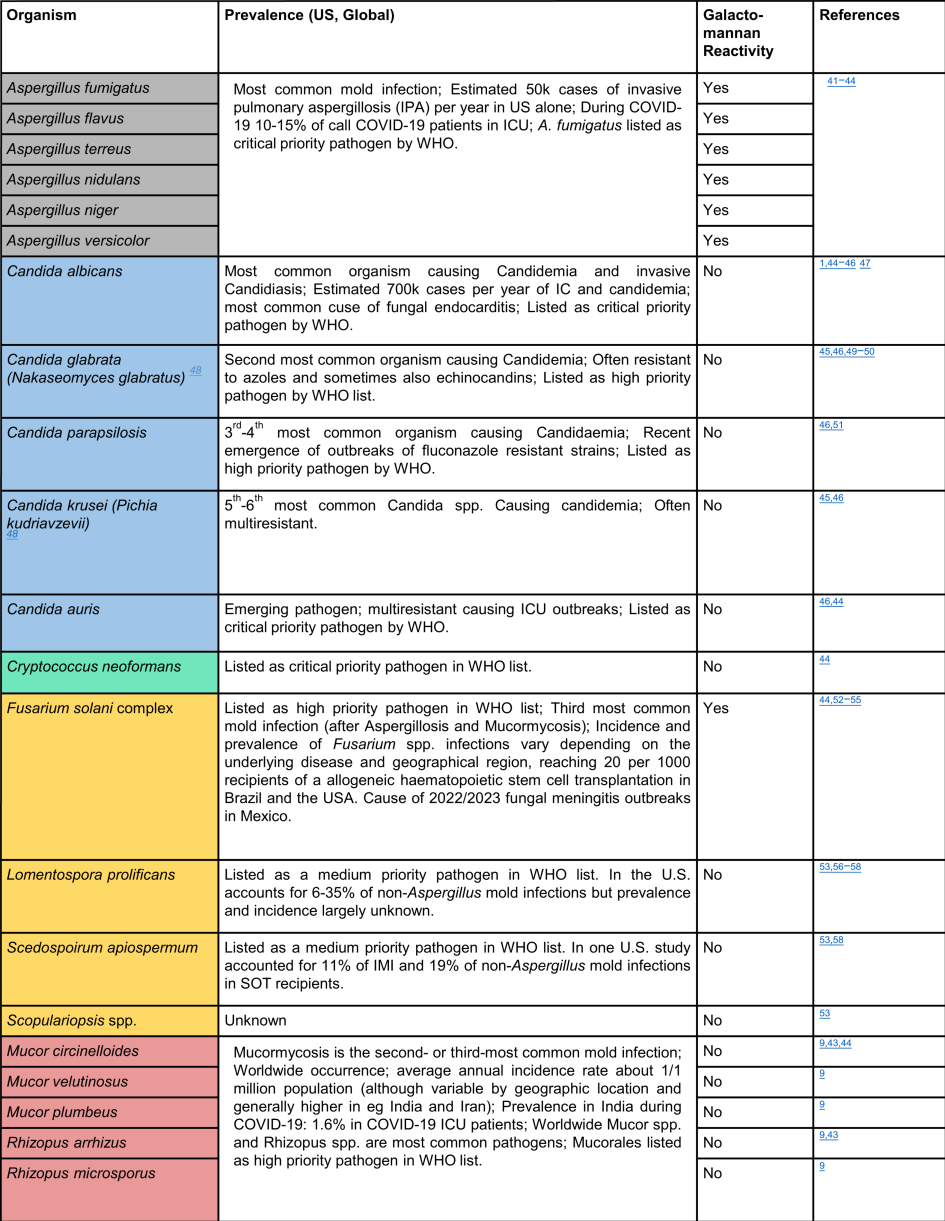
Clinically relevant fungi, including rare molds, used to develop universal assay (1, 9, 41–58).

To develop a universal PCR assay for fungal detection, we first selected primers targeting conserved sequence regions flanking the ITS1–ITS4 barcoding region of the fungal genome (Fig. S1) and tested their ability to amplify 21 clinically relevant organisms (Fig. 3). We started with *Aspergillus* spp., since it is the most prevalent IMI pathogen worldwide, and *Candida* spp., the most prevalent commensal genus, and began testing the ITS primers.

However, *Aspergillus* spp. were not consistently amplified by our ITS primers and the efficiency of this region for detection of *Aspergillus* spp. isolates is not optimal. Furthermore, the ITS1–4 region is not sufficient for discriminating between many individual *Aspergillus* spp. and has been shown to not amplify in certain isolates (59–61) (Fig. S2). Since *Aspergillus* spp. are one of the most clinically relevant fungal pathogens in the US but also globally, we next selected an *Aspergillus*-specific primer set targeting the 18S rDNA gene, which harbors species-specific sequence differences (Fig. S3).

**Fig 4:**
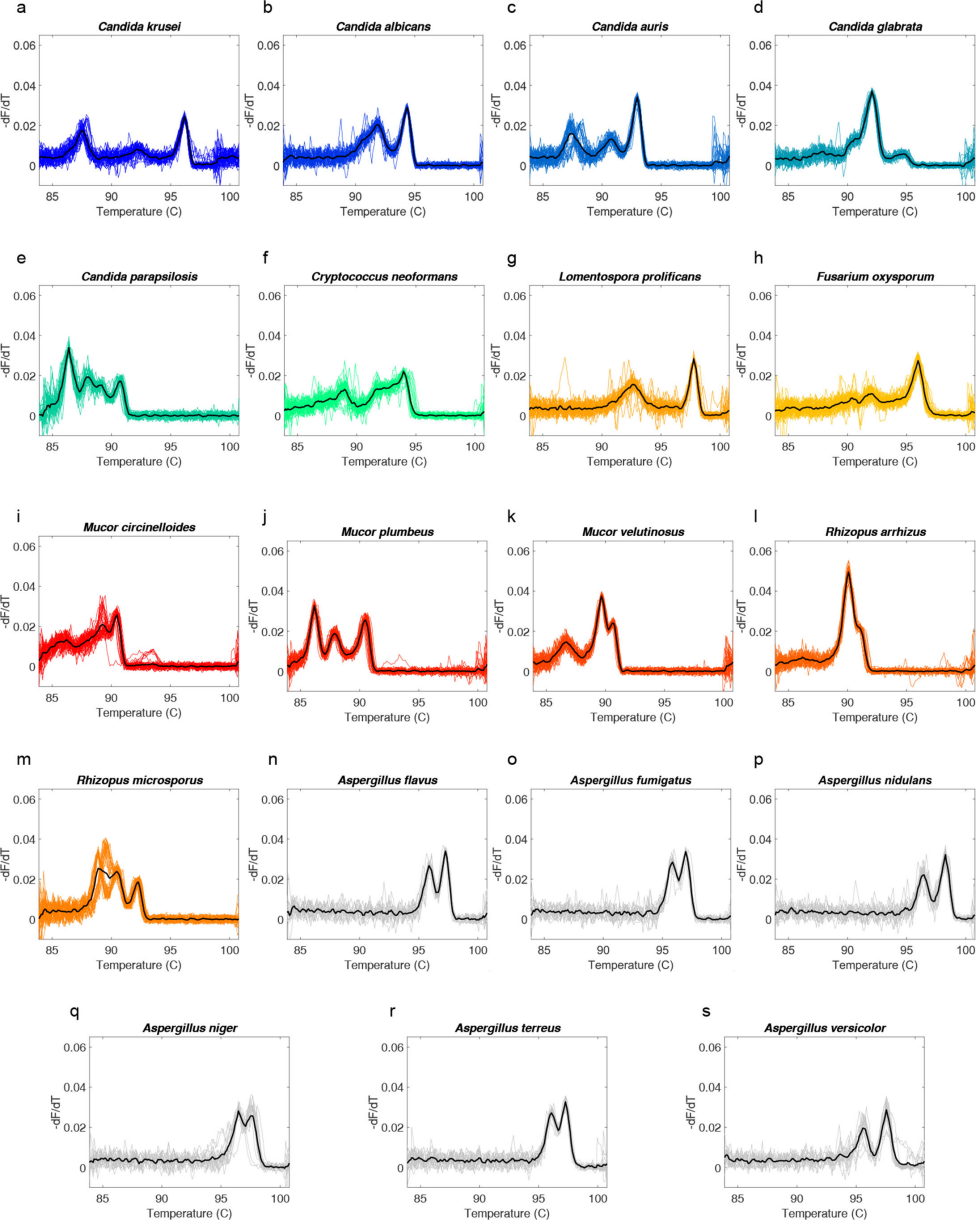
Digital melt curve database for 19 organisms. Asp and ITS primers were multiplexed in U-dHRM, and the assay was tested for the detection and melt-based discrimination of 19 organisms: (**a–e**) *Candida* spp., (**f**) *Cryptococcus* spp., (**g**) *Lomentospora* spp., (**h**) *Fusarium* spp., (**i–k**) *Mucor* spp., (**l–m**) *Rhizopus* spp., and (**n–s**) *Aspergillus* spp. Yeasts are blue/green; molds are orange/red/gray.

This primer set was multiplexed with the ITS primer set, and the assay was tested for its ability to amplify the 21 species in . Our *Scopulariopsis* spp. isolates were not consistently amplified which has been observed previously (61), while *Scedosporium apiospermum* isolates produced variable melts indicating multiple organisms (Fig. S4), and neither of these were added to the final database. Ultimately, 19 species were amplified and sequenced in qPCR and produced reliable melt curve signatures in U-dHRM. Figure 4 shows the digital melt curve signatures for each organism and their average curve in black.

Next, we conducted analytical validation studies on *Aspergillus* spp. and *Candida* spp. to assess the overall detection capability of the assay in combination with sample preparation starting from a real sample matrix. Mock samples were created by spiking whole organisms into pig BALF over a concentration range of approximately 1 × 10^5^–1×10°CFU/mL and no spike controls. Host DNA depletion and pathogen DNA extraction were carried out using MolYsis Complete5 per manufacturer’s instructions. Then, the extracted DNA was loaded onto dPCR chips with the multiplexed Asp+ITS universal fungal assay and amplification was performed prior to dHRM analysis (Fig. S5). Fungal melt curve counts showed good linearity of quantification (*r*^2^ = 0.99) for *Candida* and *Aspergillus* spp. (Fig. S6A and B). However, *Aspergillus* spp. detection was 10-fold lower than expected and Candida detection was 10-fold higher than expected, based on spore counting and plating. To test if this difference could be attributed to *Aspergillus* spp. being more difficult to lyse or whether it reflected assay sensitivity differences, we conducted *Aspergillus* spp. DNA dilution series experiments. This showed that the assay alone maintained high linearity of detection down to ~10 copies/chip or 25 pg/mL (Fig. S6C).

### Database generation and algorithm training

To determine whether fungal organism digital melt curves (Fig. 4) could be reliably and automatically recognized by a ML algorithm, a database of >150,000 curves of all combined organisms comprising biological and technical replicates *n* ≧ 3 for each of the 19 pathogens was generated on dPCR chips. Fig. 2a and b depicts the ML flowchart comparison for testing database curves versus clinical unknown or novel curves. The classification performance of a ML algorithm that combines dynamic time warping and Euclidean distance-based metrics was assessed in cross-validation studies (27).

**Fig 5:**
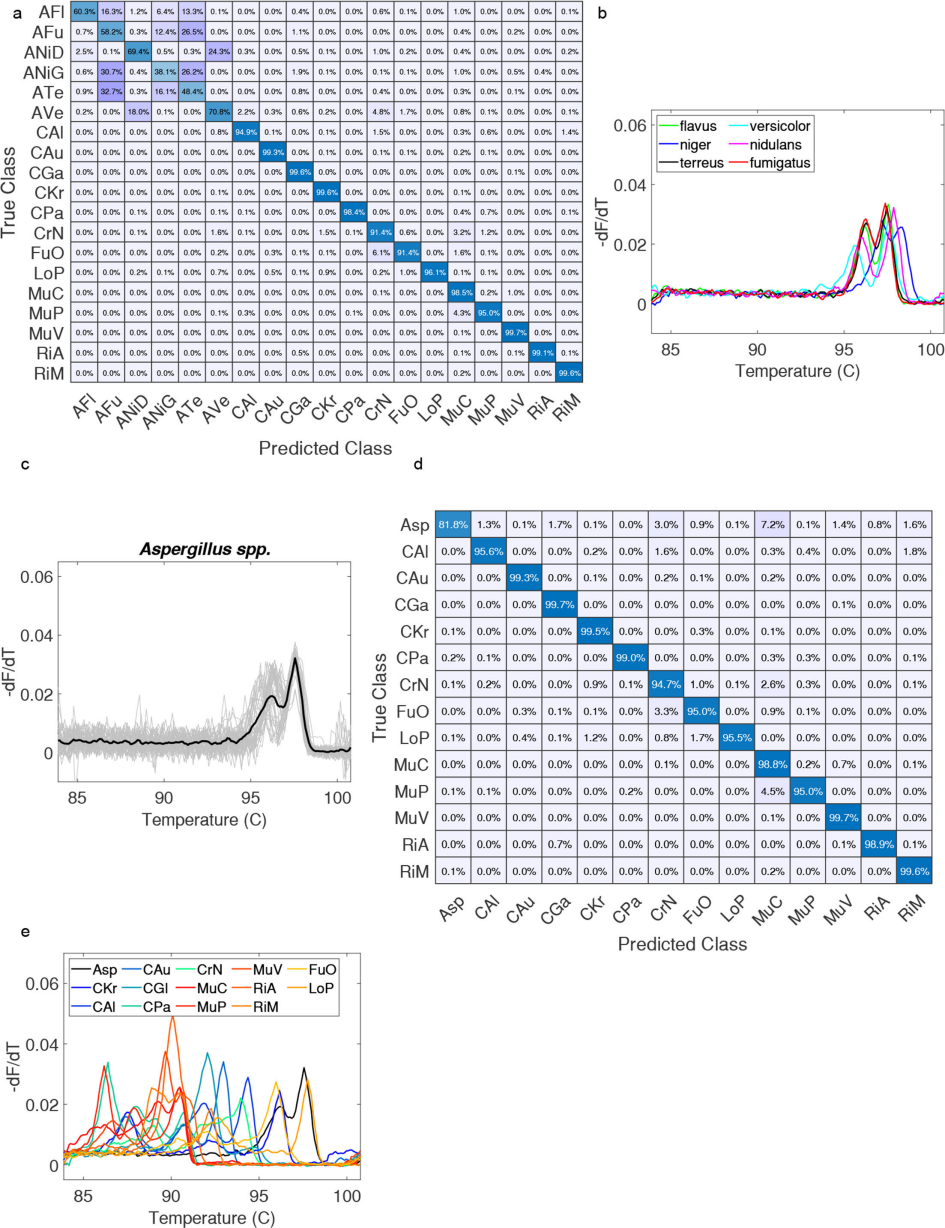
Machine classification performance on fungal melt curve database and curves. *A. flavus* (*AFl*), *A. fumigatus* (*AFu*), *A. nidulans* (*ANiD*), *A. niger* (*ANiG*), *Aspergillus terreus* (*ATe*), *Aspergillus versicolor* (*AVe*), grouped *Aspergillus* spp. (*Asp*), *C. albicans* (*C. Al*), *C. auris* (*Cau*), *C. glabrata* (*CGa*), *C. krusei* (*CKr*), *C. parapsilosis* (*CPa*), *C. Neoformans* (*CrN*), *F. oxysporum* (*FuO*), *L. prolificans* (*LoP*), *M. circinelloides* (*MuC*), *M. plumbeus* (*MuP*), *M. velutinosus* (*MuV*), *R. arrhizus* (*RiA*), and *R. microsporus* (*RiM*). (a) Confusion matrix with individual *Aspergillus* spp. (b) *Aspergillus* spp. average curves overlap. (c) Grouped *Aspergillus* spp. average curve overlap. (d) Confusion matrix with grouped *Aspergillus*. (e) Average curves of grouped *Aspergillus* genus and all average curves of 13 other spp.

Recall was assessed and plotted as a confusion matrix in Fig. 5a. This revealed that *Aspergillus* spp. were not reliably discriminated within the genus, while all other species were reliably classified. Among *Aspergillus* spp., cross-validation showed that an overall accuracy (F-score, a combination of precision and recall) of about 60% was achieved (Table S1). This can be explained visually by overlaying representative curves from each species, which are quite similar (Fig. 5b), due to few sequence differences (Fig S3). An overall accuracy of 86% was achieved across the 19 organisms with *Aspergillus* spp. treated as separate classes (Table S2). Grouping *Aspergillus* spp. into a single class (Fig. 5c) at the genus level resulted in a significant improvement in the F-score for *Aspergillus* spp. (90%, Table S3), and an overall accuracy for all classes of 97% was achieved. The associated confusion matrix (Fig. 5d) shows only 3.4% misclassification overall (5,059/150,752), with the most occurring between *M. circinelloides* and *Aspergillus* spp. when the *Aspergillus* genus is the true class (7.2%, 768/10,657). Representative melt curves for each organism class are shown in Fig. 5e.

### Clinical BALF sample analysis

#### Overall performance for pathogenic mold detection

**Fig 6:**
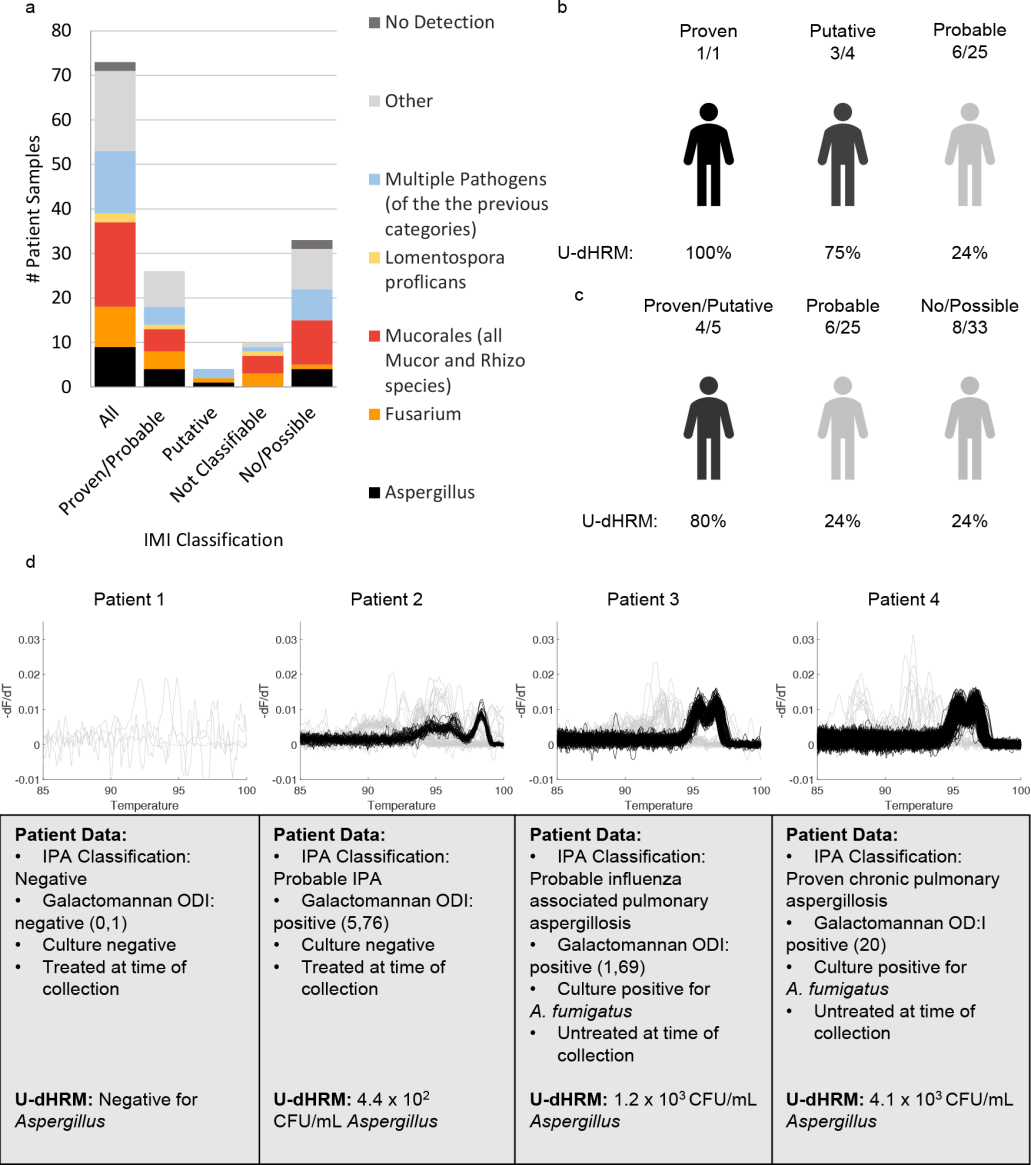
U-dHRM pathogen detection statistics in patient samples. (**a**) Pathogen distribution by IMI diagnosis classification. Others are defined as yeasts in the U-dHRM database or unknown novel organisms (b) U-dHRM detection of *Aspergillus* in suspected IMI cases ordered left to right by decreasing confidence of suspicion by IPA classification. (**c**) U-dHRM detection of *Aspergillus* in combined highest, medium, and low suspicion. (**d**) Examples of *Aspergillus* detection by U-dHRM in BALF. Concordant *Aspergillus* detection examples in patients with no IPA, probable IPA (treated at the time of collection and culture negative and untreated at the time of collection and culture positive), and proven IPA. Correlations with routine mycological test results show that more *Aspergillus* curves were detected in the patient with probable IPA who had both positive BALF GM and positive culture, versus the other patient with probable IPA who had only positive BALF GM.

SinceU-dHRM achieved an average of 97% fungal organism identification accuracy and a turnaround time of 4 hours in analytical studies, we moved forward with clinical sample studies. In total, 73 remnant-banked BALF samples that were collected due to suspicion of IMI were analyzed by U-dHRM and compared with clinical diagnostic classifications (Fig. 6). U-dHRM measured a range of fungal melt curves corresponding to 10^1^–10^5^ CFU/mL and detected pathogenic molds (*Aspergillus*, *Mucorales*, *Lomentospora*, and/or *Fusarium* spp.; ≧1 *curve* or 11 CFU/mL) in 73% (53/73) of all the samples (Fig. 6a). In addition, *Candida* spp. were detected in 88% (64/73) of all samples, while 12% (9/73) had non-*Candida* yeasts as well. We note that there was no apparent association between human β-actin cycle threshold (Ct) and concentration of fungi detected by U-dHRM or BALF sample volume and concentration of fungi detected by U-dHRM (Fig. S7). In 19% (14/73) of samples, mixtures of pathogenic molds were detected (Fig. 6a). Examples of curve signatures detected by U-dHRM and identified by ML in the clinical BALF samples and their closest matching database curve are shown in Fig. S8. Of the samples considered positive for IMI, U-dHRM detected pathogenic molds in 73% (1/1 proven, 17/25 probable, and 4/4 putative). In samples that were not classifiable for IMI, U-dHRM detected pathogenic molds in 90% (9/10). However, in samples considered negative or without mycological evidence for IMI, U-dHRM detected pathogenic molds in 67% (1/3 possible; 21/30 no). These samples were considered negative for IMI predominantly because of GM and culture negativity as well as the absence of host factors, but nonetheless, they were collected due to some clinical suspicion of IMI. These results suggest that U-dHRM has good sensitivity for IMI, as defined by the current diagnostic criteria, when host risk factors are also considered. Specificity was optimized by requiring the number of pathogenic mold curves detected in a sample to be >8 and sample volume to be 1 mL, which resulted in a subset of 43% detection in criteria-matching positives (6/14), 50% (5/10) in not classifiable, and 0% detection in negatives (0/21) .

#### *Aspergillus* detection by U-dHRM compared with culture and GM

A summary of *Aspergillus* spp. detection by U-dHRM compared with clinical diagnostic criteria is shown in Fig. 6b and c. Of all the samples that cultured *Aspergillus* spp., U-dHRM detected *Aspergillus* spp. melt curves in 61% of positives (1/1 proven, 4/9 probable, and 3/3 putative), 0% of not-classifiable (0/2) cases, or no IPA (0/1 no). Considering only samples from proven, probable, and putative cases that were culture+, GM+, and antifungal treatment−, U-dHRM detected *Aspergillus* spp. melt curves in 78% (7/9). Examples of *Aspergillus* spp. melt curves from patient samples that correlated with routine mycological test results are shown in Fig. 6d. The highest *Aspergillus* spp. load was detected in the patient with proven IPA, and the second highest load was detected in a patient with probable influenza-associated pulmonary aspergillosis.

U-dHRM also detected *Aspergillus* spp. in some samples that did not culture *Aspergillus* spp.: 10% (2/19) probable, 12% (1/8) not classifiable, and 28% (8/29) no IPA. In samples that did not culture *Aspergillus* spp., other pathogenic molds were often detected by U-dHRM alone or in combination with *Aspergillus* spp.: other molds were detected in 71% of probable and putative cases (12/17), 70% (7/10) not classifiable cases, and 67% (22/33) of possible and no IPA cases.

#### Differentiation between *Aspergillus* spp. and *Fusarium* spp. by U-dHRM in GM-positive samples

Of all the GM+ samples, U-dHRM detected GM-producing organisms *Aspergillus* and/or *Fusarium* spp. in 54% (21/39). Mixtures of *Aspergillus* and *Fusarium* spp. were detected in 8% (3/39).

**Fig 7:**
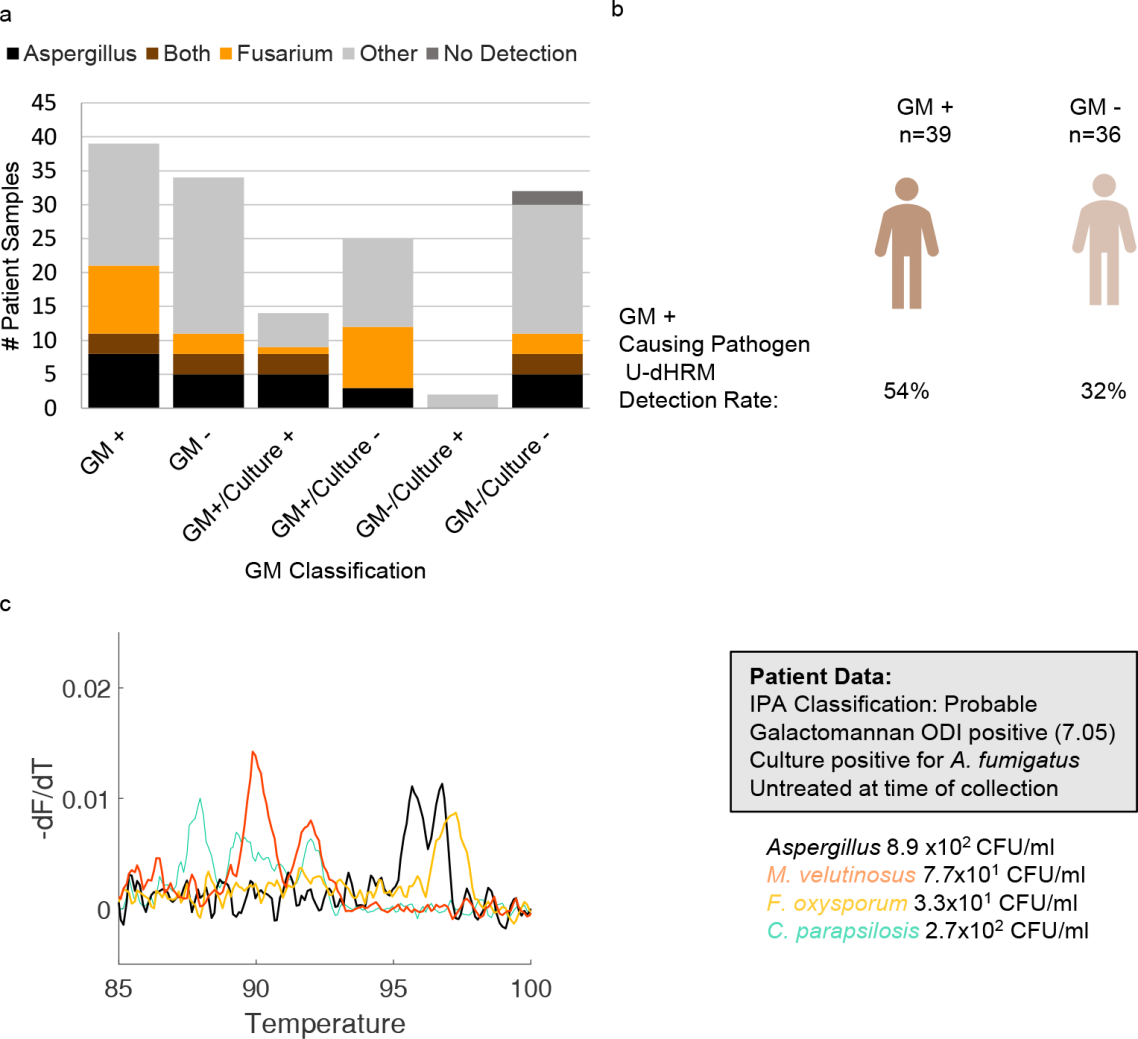
*Aspergillus* and *Fusarium* co-detection. (**a**) *Aspergillus* and *Fusarium* detection distribution by GM and culture positivity. Others are defined as yeasts in the U-dHRM database or unknown novel organisms. (b) U-dHRM detection of GM-producing spp. compared with clinical GM status. (**c**) Representative raw melt curves from a clinical sample where *Aspergillus*, *Mucor*, *Fusarium*, and *Candida* were co-detected. Organism quantification by U-dHRM was: 8.9 × 10^2^ CFU/mL *Aspergillus*, 3.3 × 10^1^ CFU/mL *F. oxysporum*, 7.7 × 10^1^ CFU/mL *M. veluntunsosis*, 2.7 × 10^2^ CFU/mL *C. parapsilosis*, and 3.1 × 10^2^ CFU/mL novel organisms (not shown).

In GM+/*Aspergillus* spp. culture+ samples, *Aspergillus* spp. alone were detected in 36% (5/14) and *Fusarium* spp. alone in 7% (1/14), while both were detected in 21% (3/14). In GM+/*Aspergillus* spp. culture- samples, *Aspergillus* spp. were detected in 12% (3/25) and *Fusarium* spp. in 36% (9/25), while both were detected in 0% (0/25). These results are depicted in Fig. 7a and b. An example of multiple pathogen detection including *Aspergillus* and *Fusarium* spp. melt curves from a patient sample is shown in Fig. 7c.

#### Detection of pathogenic molds in the absence of *Aspergillus*

In samples where no *Aspergillus* spp. was detected by U-dHRM, other pathogenic molds were detected in putative 1/1 (100%), probable 11/19 (58%), not classifiable 89% (8/9), possible 33% (1/3), and no IMI 55% (12/22) cases.

#### *Mucorales* detection

Fungal pathogens in the *Mucorales* order were detected in 42% (31/73) of all samples. *Mucorales* was detected in 31% (8/26) of the proven/probable IMI cases, 50% (2/4) putative cases, 40% (4/10) not classifiable cases, 33% (1/3) of the possible cases, and 53% (16/30) of the samples classified as no IMI. Under optimal specificity criteria (>8 pathogenic mold curves detected and sample volume at least 1mL), this subset of detection dropped to 15% (2/13) in proven/probable IMI cases, 100% (1/1) of putative cases, 30% (3/10) of not classifiable cases, and no detection in possible cases or samples classified as no IMI.

**Fig 8:**
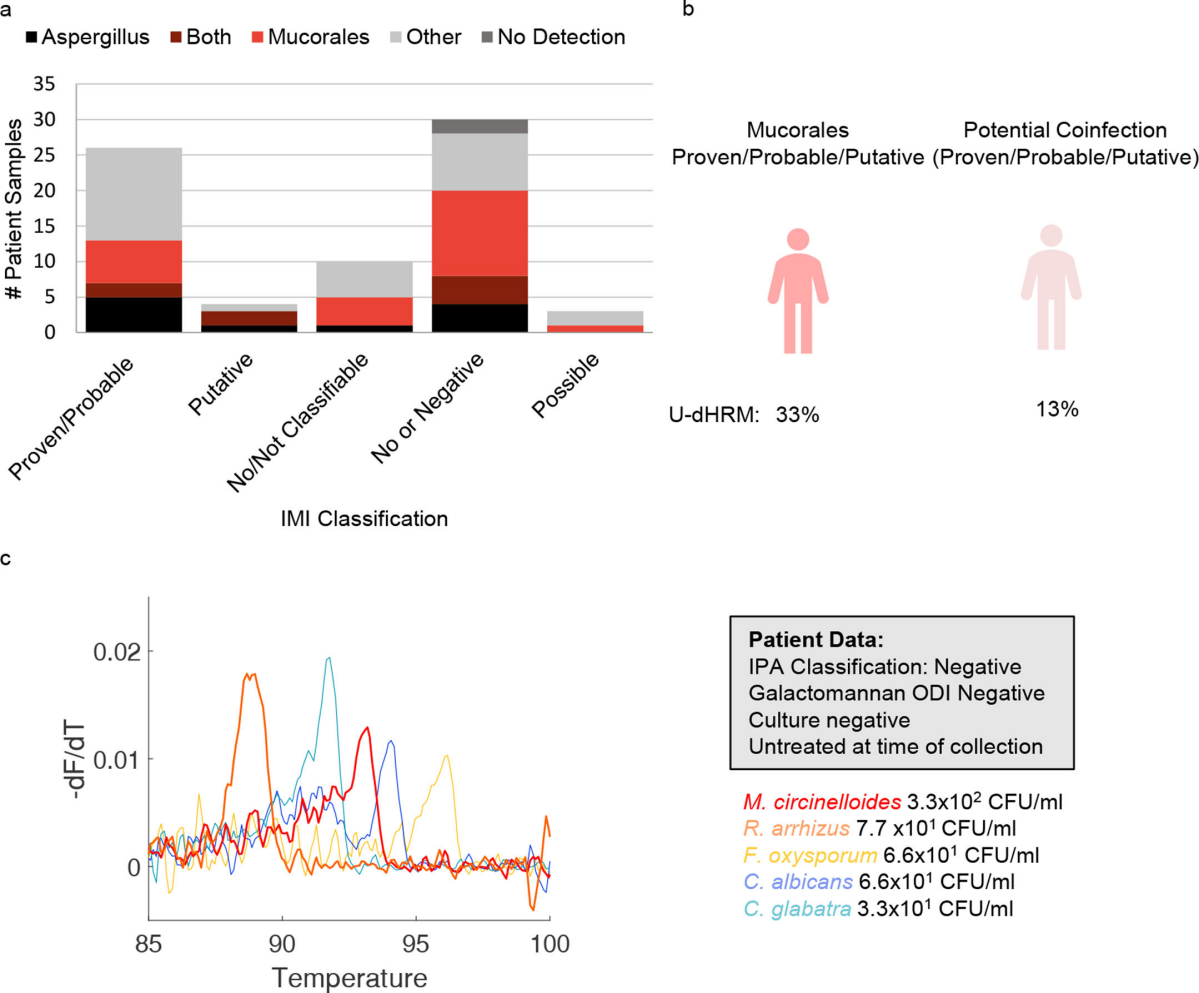
*Mucorales* detection. (a) *Aspergillus* and *Mucorales* detection distribution by IMI diagnosis classification. Others are defined as fungi and yeasts in the U-dHRM database or unknown novel organisms. (b) U-dHRM detection of *Mucorales* and potential co-infection in proven, probable, and putative IMI cases. (**c**) Discordant mold diagnosis example showing representative raw melt curves from patient BALF sample. Curves are shown for *Mucor*, *Fusarium*, and *Candida* for visualization purposes with the following quantifications: 6.6 × 10^1^ CFU/mL *C. albicans* (blue), 3.3 × 10^1^ CFU/mL *C. glabrata* (blue), 3.3 × 10^2^ CFU/mL *M. circinelloides* (red), 7.7 × 10^1^ CFU/mL *R. arrhizus* (orange), 6.6 × 10^1^ CFU/mL *F. oxysporum*, and 1.2 × 10^2^ novel organisms (not shown).

Co-detection of ≧1 curve for multiple *Mucorales* spp. occurred in 10% (7/73) of samples, with the highest rate in possible at 33% (1/3), followed by not classifiable at 20% (2/10), no IMI at 10% (3/30), and probable at 4% (1/25) , with proven and possible at 0%. Co-detection of *Mucorales* and *Aspergillus* spp. occurred in 11% (8/73) of samples, with the highest rate in putative at 50% (2/4) followed by samples classified as no IMI at 13% (4/30), and proven/probable at 8% (2/26), with no co-detection in those classified as possible and those classified as not classifiable. These results are depicted in Fig. 8a and b. An example of co-detection of *Mucorales* spp., including melt curves from a patient sample representing discordant mold diagnosis, is shown in Fig. 8c.

#### Identification of organisms generating novel fungal melt curves

**Fig 9:**
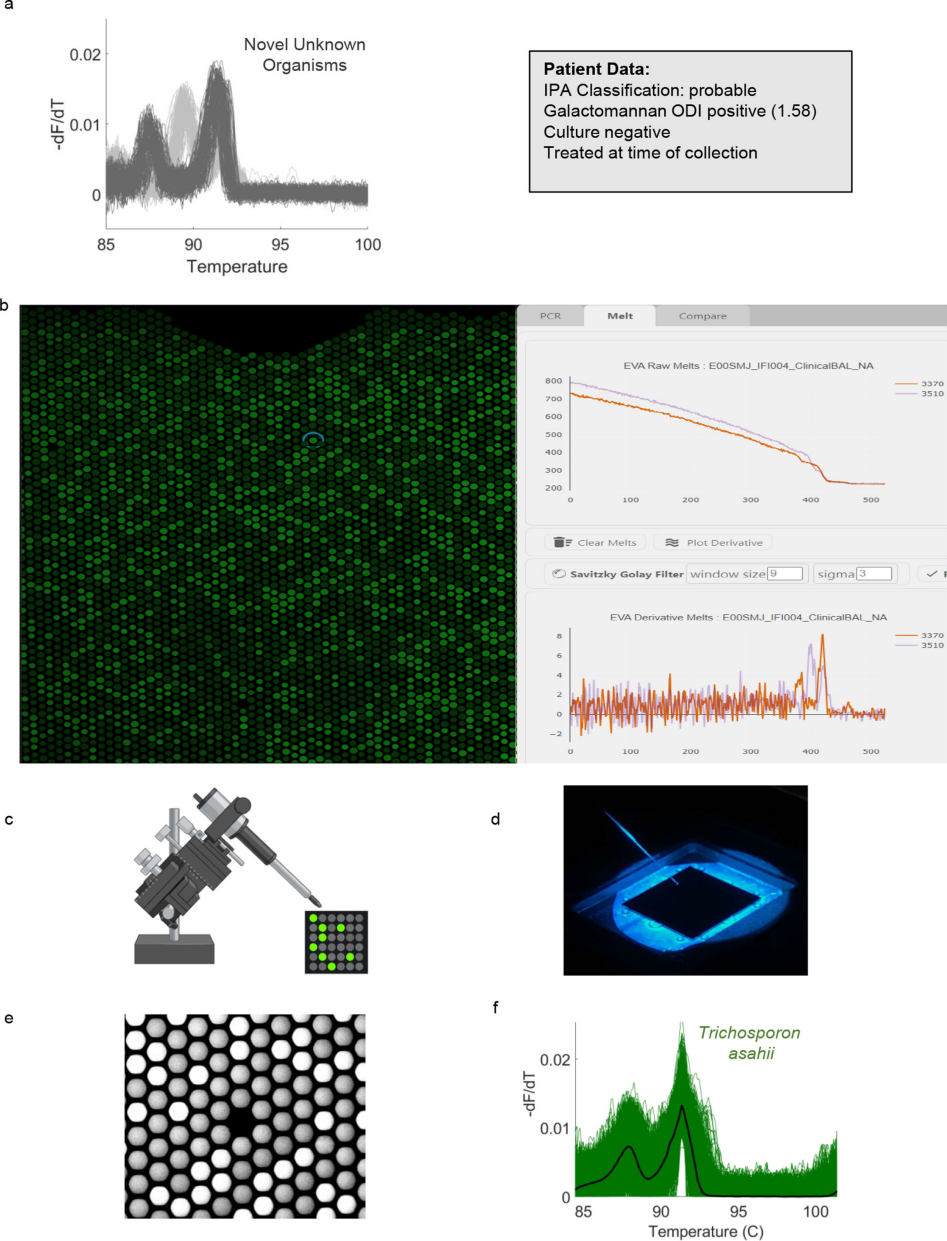
Novel melt curve identification and algorithm retraining. (**a**) Novel fungal melt curves *Trichosporon asahii* (dark-gray) and *Saccharomyces cerevisiae* (light-gray) identified by ML for patient sample IFI 004. Diagnostic information for this patient is shown in the adjacent gray box. (**b**) Screenshot of the Melio Melt Inspector software used to find the specific XY location of the wells on-chip harboring novel amplicons. (**c and d**) Schematic and photograph of the micromanipulator positioning a micropipette into the target well for novel amplicon collection. (**e**) Fluorescent micrograph of chip after micropipette extraction of reaction from the target well. (**f**) U-dHRM melt curves generated by re-amplification of the novel amplicon for database expansion and training of the ML algorithm.

A unique feature of the U-dHRM-trained ML algorithm is its ability to automatically detect novel organisms by their distinct melt curve shapes compared with common pathogen curves represented in the database (see Materials and Methods). Ninety-six percent (70/73) of the BALF samples tested produced melt curves that confidently matched to the U-dHRM database of common pathogens. However, a few patient samples generated fungal melt curves that did not match the database and were called novel by the algorithm. To identify the organisms generating these curves, a micromanipulator was used to recover individual digital reactions and sequence their amplicons. Figure 9 demonstrates the application of this new technique to a patient sample where novel melt curves dominated U-dHRM results (Fig. 9a). Custom software was used to determine the XY position of novel curve-generating wells (Fig. 9b), and wells were sampled by using a micromanipulator (Fig. 9c) to position a micropipette into the target well (Fig. 9d) and extract the reaction containing novel amplicons (Fig. 9e). In this sample, *Trichosporon asahii* and *Saccharomyces cerevisiae* (Fig. 9a, dark- and light-gray curves, respectively) were identified. Using the recovered *T. asahii* amplicons as template, U-dHRM was conducted to generate database curves for training the ML algorithm to automatically identify this organism in future samples Fig. 9f. Table S4 describes other patient samples where novel amplicons were recovered and identified, including potentially causative pathogens and commensal yeasts *Pneumocystis jirovecii*, *Sporobolomyces salmonicolor*, *Saccharomyces cerevisiae*, *Epicoccum nigrum*, and *Candida inconspicua*. This process avoids the need to culture amplify isolates, which is important considering the low sensitivity of BALF culture and potential fastidiousness of novel organisms. Additionally, it will further expand the database while limiting the occurrence of future unidentifiable melt curves, thus minimizing the need for future sequencing, which in turn affects the TAT.

## DISCUSSION

In this study, all patients who had BALF collected were suspected of having fungal infection, and it was determined necessary to order GM and culture testing. U-dHRM detected potential fungal pathogens in 73% of 30 samples classified as positive (proven, probable, or putative) for IMI, including mixed infections. However, it also detected potential fungal pathogens in 67% of cases considered negative (possible or no) IMI. Specificity was optimized by requiring the number of pathogenic mold curves detected in a sample to be >8 and a sample volume to be 1 mL, which resulted in 100% specificity in 21 at-risk patients without IMI. U-dHRM also showed high sensitivity and specificity in analytical validation experiments. One explanation for the seemingly high false positive rate of detection by U-dHRM is that the presence of an organism could indicate infection or could represent colonization or components of the lung mycobiome. Quantitative melt curve cutoffs may be useful to distinguish infection from colonization and could be used for monitoring of organism loads and community proportions. The fact that evaluating the performance of new diagnostic tests for IMI is difficult may have also contributed to the discrepancy, arising from the limitations of comparing these new modalities to imperfect gold standard clinical tests, the rarity of autopsy-proven IMI, and the ongoing debate over the accuracy of diagnostic classifications (62). For example, the sensitivity of culture from BALF has been reported to range from 30% to 60%, even in patients with proven *Aspergillus* pulmonary infection (63, 64), while meta-analysis for the sensitivity of GM testing in BALF reports a range of 78%–88% (65). Combining culture and GM tests in an “and” manner results in an overall sensitivity of 23%–53%. Imperfect gold standard tests can contribute to an appearance of high false positives in new tests.

U-dHRM results were not particularly well correlated with GM positivity or *Aspergillus* spp. culture results, neither of which correlated well with each other. However, U-dHRM did demonstrate strong agreement with clinical mycology tests in general and showed good sensitivity for IMI, as defined by current diagnostic criteria, when host factors were also considered. When considering samples from proven, probable, and putative cases that were culture+, GM+, and antifungal treatment−, U-dHRM performed well, detecting *Aspergillus* spp. melt curves in 78% (7/9). A significant proportion (43% 13/30) of proven, probable, and putative IPA patients were on antifungal therapy at the time of collection, which might have decreased the number of organisms present in the BALF fluid, thus impairing identification by U-dHRM. It has been posited in other fungal PCR studies that highly potent antifungal therapy and new prophylactic treatment schemata’s may reduce fungal load to undetectable amounts in the extravascular compartments (66).

U-dHRM detected *Aspergillus* spp. in 61% (9/13) of culture-positive samples from patients with IPA; the method also detected *Aspergillus* spp. in 19% (11/58) of culture-negative samples. Only 9% of total eluted DNA from each clinical sample was analyzed, which could have impacted the assay’s sensitivity. The presence of viable but non-culturable organisms may also explain this finding. MolYsis sample processing upstream of U-dHRM analysis utilizes selective lysis, DNase, and filtration steps to degrade host and cell-free DNA and enrich for intact organisms, which allows U-dHRM to detect organisms that are intact but may not grow in culture. The sample processing may have also contributed to some discrepancies, since culture was conducted at the time of sampling but U-dHRM was conducted after samples had been frozen and stored for up to 8 years. Freezing and long storage may have led to organism lysis, and DNA degradation prior to sample preparation and MolYsis treatment, which degrades cell-free DNA, could have contributed to missed detections by U-dHRM, explaining some of the negative results in patients with prior *Aspergillus* detection by culture. Analytical study results also suggest that the lysis step prior to U-dHRM could be improved to facilitate higher sensitivity for difficult-to-lyse organisms like *Aspergillus* spp. (67). One such example would be to include intensive bead beating, as has been developed by the European Aspergillus PCR Initiative for the extraction of *Aspergillus* DNA from whole blood, serum, and plasma (68). An additional consideration for discrepancies between U-dHRM and GM results is the possibility of organism clearance when antigen levels are high or the presence of organisms before antigens are developed during active growth. Importantly, in cases where GM positivity did not correlate with *Aspergillus* spp. detection by culture, U-dHRM results occasionally provided potential explanations by detecting other GM-producing organisms such as *Fusarium* and *Trichosporon* spp. (69).

With BALF culture showing limited sensitivity for detecting pulmonary fusariosis (51), *Fusarium* spp. infections resulting in GM positivity can lead to a false diagnosis of probable IPA and incorrect or inadequate antifungal treatment for these highly resistant pathogens. In San Diego, *Fusarium* spp. have been shown to be a frequent cause of rare mold infections (55). U-dHRM had higher detection of *Fusarium* in GM+ samples that did not grow *Aspergillus* spp. in culture. Also, the ability of U-dHRM to detect multiple common pathogens, even in mixtures, has potential to identify mixed infections and improve treatment decisions. For example, in a patient classified as probable for IPA with positive GM and *Aspergillus* spp. culture results, U-dHRM detected *Aspergillus* spp. in concordance with these results but also detected *F. oxysporum* and *M. velutinosus* at similar abundances (8.9 × 10^2^ CFU/mL *Aspergillus*, 3.3 × 10^1^ *F*. *oxysporum*, 7.7 × 10^1^ CFU/mL *M*. *velutinosus*, and 2.7 × 10^2^ CFU/mL *C*. *parapsilosis*). While at the time of BALF collection, this patient had not received antifungal treatment, the patient subsequently received treatment for IPA with voriconazole (which likely covered *F. oxysporum* but not *M. velutinosus*) and passed away within a week, with no autopsy performed. In this case, U-dHRM results may have influenced treatment to include antifungals targeting *M. velutinosus*. In another example, U-dHRM detected a mixture of different *Mucorales* spp. in a patient with suspected IMI but negative GM and *Aspergillus* spp. culture results. Of note, one of the species detected, *M. circinelloides*, commonly shows higher MICs against isavuconazole and posaconazole, complicating therapy (70). While IPA can be diagnosed with the presence of host factors, clinical symptoms, radiological findings, and mycological evidence of *Aspergillus* either in culture or by detection of GM, other IMIs can mimic the clinical presentation of IPA, with mycological evidence mostly limited to insensitive culture or histology.

U-dHRM yielded a high positive rate (53%) for *Mucorales* in BALF from patients determined to be negative for IMI. However, clinical diagnosis relied heavily on culture (no PCR was used), which often yields false negative results from patients with mucormycosis because of hypha fragmentation during sample processing (71). Mucormycosis is the most frequent rare mold infection in San Diego (55), and reported local rates were based on culture only. It is therefore likely that many people in San Diego are exposed to some extent. Implementing a curve number threshold in U-dHRM to remove all *Mucorales* detection in the negative population left six cases in other diagnostic categories that exceeded the cutoff (two probable, one putative, and three not classifiable cases). Quantitative cutoffs and monitoring over time therefore seem necessary as U-dHRM detection in BALF may not always indicate infection but also colonization/components of the lung mycobiome. Even the detection of *Mucorales* as a colonizing agent could inform treatment decisions. For example, in a patient with *Aspergillosis* infection who also has *Mucorales* colonization, likely an antifungal agent that covers *Mucorales* at least to some extent would be selected.

The ability of U-dHRM to detect novel fungal organisms also demonstrated diagnostic value in this patient cohort. Several patient samples contained more novel melt curves than curves from common pathogens. The ability to recover these amplicons for same-day Sanger sequencing enabled the fast identification of emerging pathogens of clinical significance. In one case, using this method resulted in the identification of *T. asahii* as the dominant organism in the BALF of a patient classified as probable for IPA with positive GM and negative culture who had already received 42 days of micafungin. U-dHRM did not detect *Aspergillus* spp. in that patient. *T. asahii* is resistant to micafungin and can cause positive GM. It is an emerging pathogen that is rarely identified in clinical practice but often causes fatal infections in immunocompromised individuals due to being misdiagnosed as other types of fungal infections and because of its resistance to many front-line antifungals (72). This particular patient was never diagnosed with or treated for *T. asahii* and passed away, suggesting that U-dHRM could have provided critical diagnostic value with high impact for this patient.

Overall, the performance of U-dHRM suggests that it could represent a promising advance in molecular pathogen detection strategies for IMI. Previously, broad-based qPCR followed by sequencing has shown promise for improving the detection of rare molds, but this approach is recommended *only* when fungal elements are seen by histopathology due to sensitivity limitations (34). Also, the presence of multiple fungal species can lead to the detection of only the dominant species or failed detection altogether (73, 74). U-dHRM distinguishes itself by implementing broad-based PCR in a higher sensitivity dPCR format. Implementation of melt analysis in a digital format enables identification and counting at the single genome level, even in polymicrobial samples, and eliminates template amplification competition and efficiency biases. This format allows extensive melt curve training data to be rapidly generated, unlocking the power of machine learning through big data for automated melt curve identification to rapidly identify and quantify the sequences of all the common pathogens in the sample individually. Only novel organism curves of high abundance warrant interrogation by sequencing, saving time and expense. U-dHRM technology allows for a broader snapshot of the patient pathobiome, including more sensitively detecting and discriminating causative species. The quantitative nature of U-dHRM results also highlight the potential for monitoring over time to track mixed infections, measure effectiveness of therapies, and aid in discriminating between true infection (growth) and colonization (stasis).

Based on total curve counts per chip and Poisson theory, we estimate that approximately 10% of samples (7/73) had 1.6% of total wells with double occupancy. So multiple organism curves could have overlapped in these wells, which may generate multiplexed curves that would be called novel. Running U-dHRM on a dilution of these samples overcomes this challenge. ML could also be potentially trained on combined melt curves in multiple occupancy wells. Also, *Aspergillus* spp. curves were not reliably differentiable, indicating that the sequence diversity of the *Aspergillus* specific amplicon generated by the selected primers was not sufficient. Future studies should re-engineer the assay to ensure sufficient sequence diversity to yield distinguishable melt curve shapes. For example, the β-tubulin gene may offer a promising alternative for suptyping *Aspergillus* and *Scedosporium* (60).

### Conclusions

The promising performance and speed of U-dHRM and its ability to simultaneously identify and quantify clinically relevant mold pathogens in polymicrobial samples as well as detect emerging opportunistic pathogens may provide information that could aid in treatment decisions and improve patient outcomes. For BALF, U-dHRM may be useful as a parallel test in high-prevalence areas/populations to maximize sensitivity or as a test conducted serially after other tests in areas/populations of low prevalence to maximize specificity. Future studies will be run on freshly obtained BALF samples instead of remnant banked samples to further evaluate the sensitivity of U-dHRM as well as the host depletion influence on possible loss of microbial reads. Concurrent BALF and blood samples will be assessed to provide to help discriminate angioinvasive infections (75). Sampling from timepoints before and after IMI classification would aid in evaluating U-dHRM’s diagnostic power compared with the gold standard tests, diagnostic classifications, and response to treatment. While our study has shown the potential of this method to aid IMI diagnosis, all these measures could also help to establish a reliable cutoff for improving specificity for infection versus colonization and thereby accuracy.

## SUPPLEMENTAL MATERIAL

Supplemental material jcm.01476-23-s0001.docx Tables S1 to S4; Figures S1 to S9.

ASM does not own the copyrights to Supplemental Material that may be linked to, or accessed through, an article. The authors have granted ASM a non-exclusive, world-wide license to publish the Supplemental Material files. Please contact the corresponding author directly for reuse.
