# Supplementary material for: Universal digital high-resolution melting for the detection of pulmonary mold infections
Source: J Clin Microbiol. 2024 May 2;62(6):e01476-23. doi: 10.1128/jcm.01476-23 (PMC11237519; doi:10.1128/jcm.01476-23)
Supplement: Supplemental material — Tables S1 to S4; Figures S1 to S9. [file jcm.01476-23-s0001.docx]

**List of Supplementary Materials**

**Supplementary Tables**

Table S1. Classification performance of *Aspergillus* spp. alone as separate classes

Table S2. Classification performance of *Aspergillus* spp. alone as separate classes vs. full database

Table S3. Classification performance of *Aspergillus* spp. grouped vs. full database

Table S4. Full patient sample U-dhRM breakdown

**Supplementary Figures**

Fig. S1. ITS primer alignment

Fig. S2. dPCR amplification of *C. albicans* and *A. fumigatus* using ITS primers only

Fig. S3. *Aspergillus* specific primer alignment

Fig S4. Excluded organisms

Fig S5. U-dHRM process flow

Fig S6. Analytical Validation Testing

Fig S7. Comparison of human β-actin Ct and concentration of fungi detected or sample volume and concentration of fungi

Fig S8. Examples of clinical curve clusters matching database averaged clusters

Fig S9. Plate counts vs. spike in, Aspergillus picogreen dilution

**SUPPLEMENTARY MATERIALS**


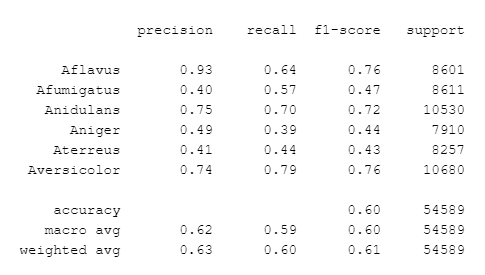


**Supplementary Table 1.** Classification performance in cross-validation studies of *Aspergillus* spp. alone as separate classes. Support column indicates the number of melt curves.


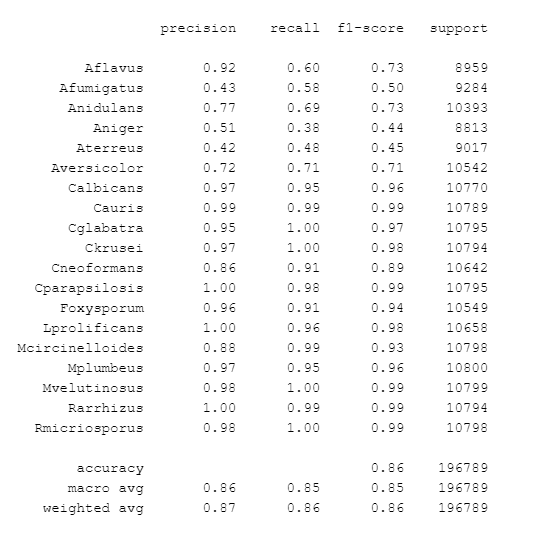


**Supplementary Table 2.** Classification performance in cross-validation studies where *Aspergillus* spp. are separate classes. Support column indicates the number of melt curves.


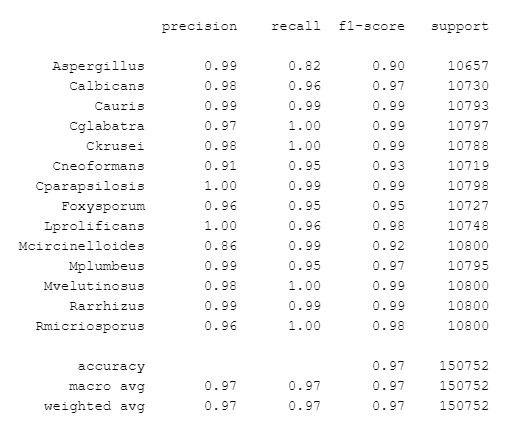


**Supplementary Table 3.** Classification performance in cross-validation studies where Aspergillus spp. were grouped as a single genus class. Support column indicates the number of melt curves.

| Sample ID | Sample Volume  Tested (ul) | Final diagnosis IPA classification | Aspergillus Culture Positivity | GM | Treatment | Any Fungi U-dHRM + | Aspergillus U-dHRM + | Novel Organisms Detected by Micromanipulator | Human  Cq (Avg) |
| --- | --- | --- | --- | --- | --- | --- | --- | --- | --- |
| H051 | 1000 | proven | *A. fumigatus* | 20 | - | + | + |  | 24.87 |
| H003 | 900 | probable | *A. fumigatus* | 7.05 | - | + | + |  | 25.89 |
| H008 | 850 | probable | *A. fumigatus* | 9.6 | + | + | - | *Pneumonocystis jirovecii*  *Sporobolomyces salminocolor* | 28.76 |
| H040 | 625 | probable | *A. fumigatus* | 1.69 | - | + | + |  | 27.74 |
| H041 | 1000 | probable | - | 5.84 | + | + | - |  | 24.11 |
| H05 | 1000 | probable | - | 5.08 | - | + | - |  | 25.87 |
| H054 | 1000 | probable | - | 3.09 | + | + | - |  | 41.93 |
| H056 | 1000 | probable | *A. fumigatus* | 0.77 | + | + | + |  | 23.87 |
| H057 | 1000 | probable | - | 1.1 | + | + | - |  | 20.81 |
| H064 | 1000 | probable | - | 3.8 | - | + | - |  | 22.37 |
| H066 | 1000 | probable | - | 5.73 | + | + | - |  | 13.34 |
| H070 | 1000 | probable | - | 5.76 | + | + | + |  | 17.98 |
| H078 | 1000 | probable | - | 6.04 | + | + | - |  | 30.09 |
| H10 | 1000 | probable | - | 5.92 | + | + | - |  | 26.56 |
| H13 | 950 | probable | - | 9.76 | + | + | - |  | 27.28 |
| H16 | 865 | probable | *A. nidulans* | 1.48 | + | + | - |  | 23.5 |
| H29 | 500 | probable | - | 1.68 | + | + | - |  | 26.17 |
| IFI123 | 1000 | probable | *A. fumigatus* | 0.1 | - | + | - |  | 22.12 |
| IFI  R007 | 690 | probable | *A. terreus* | 1.35 | - | + | - |  | 25.12 |
| IFI001 | 1000 | probable | - | 1.45 | - | + | - |  | 23.48 |
| IFI004 | 800 | probable | - | 1.58 | + | + | - | *Sacchromyces cerevisae*  *Trichosporon*  *asahii* | 25.31 |
| IFI037 | 500 | probable | - | 1.29 | - | + | - |  | 29.45 |
| IFI043 | 675 | probable | - | 2.82 | - | + | + |  | 26.1 |
| IFI047 | 375 | probable | - | 2 | - | + | - |  | 30.05 |
| R08 | 690 | probable | *A. versicolor* | 5.36 | - | + | - |  | 21.57 |
| R18 | 900 | probable | *A. fumigatus* | 1.75 | - | + | + |  | 23.58 |
| H14 | 1000 | putative | *A. fumigatus* | 4.35 | - | + | + |  | 28.52 |
| H24 | 420 | putative | *A. nidulans* | 11.93 | - | + | + |  | 24.91 |
| IFI056 | 800 | putative | - | 5.08 | - | + | - |  | 27.36 |
| R11 | 950 | putative | *A. fumigatus* | 5.4 | - | + | + |  | 37.7 |
| IFI061 | 1000 | possible | - | 0.1 | - | + | - |  | 30.61 |
| IFI114 | 1000 | possible | - | 0.1 | - | + | - |  | 24.02 |
| IFI118 | 1000 | possible | - | 0.1 | - | + | - |  | 25.05 |
| H002 | 650 | no | *A. fumigatus* | 6.83 | - | + | - |  | 27.63 |
| H042 | 1000 | no | - | 0.67 | - | + | + |  | 32.37 |
| H049 | 1000 | no | - | 0.91 | - | + | - |  | 29.5 |
| H050 | 1000 | no | - | 0.87 | - | + | - |  | 33.57 |
| H052 | 1000 | no | - | 0.79 | - | + | - |  | 30.32 |
| H055 | 1000 | no | - | 0.85 | - | + | - |  | 28.66 |
| IFI062 | 500 | no | - | 0.1 | - | + | - |  | 25.65 |
| IFI063 | 800 | no | - | 0.1 | - | + | + |  | 25.07 |
| IFI064 | 1000 | no | - | 0.1 | - | + | - | *Epicoccum nigrum* | 24.45 |
| IFI069 | 500 | no | - | 0.1 | - | + | + |  | 28.59 |
| IFI071 | 1000 | no | - | 0.1 | - | + | - |  | 23.93 |
| IFI072 | 750 | no | - | 0.1 | - | + | + |  | 24.12 |
| IFI074 | 1000 | no | - | 0.1 | - | + | - |  | 26.8 |
| IFI075 | 900 | no | - | 0.1 | + | + | - |  | 27.64 |
| IFI077 | 1000 | no | - | 0.1 | - | + | - |  | 25.33 |
| IFI079 | 1000 | no | - | 0.1 | - | + | + |  | 20.31 |
| IFI080 | 1000 | no | - | 0.1 | - | + | - |  | 27.74 |
| IFI084 | 950 | no | - | 0.1 | - | + | - |  | 25.5 |
| IFI085 | 860 | no | - | 0.1 | - | + | - |  | 28.99 |
| IFI087 | 1000 | no | - | 0.1 | - | + | + |  | 26.19 |
| IFI088 | 550 | no | - | 0.1 | - | + | - |  | 23.7 |
| IFI089 | 1000 | no | - | 0.1 | - | - | - |  | 25.62 |
| IFI098 | 1000 | no | - | 0.1 | - | + | - |  | 22.82 |
| IFI107 | 1000 | no | - | 0.1 | - | + | - |  | 24.41 |
| IFI116 | 1000 | no | - | 0.1 | + | + | - |  | 23.49 |
| IFI124 | 1000 | no | - | 0.1 | - | - | - |  | 24.03 |
| IFI094 | 1000 | no | - | 0.1 | - | + | - |  | 21.8 |
| IFI099 | 800 | no | - | 0.1 | - | + | - |  | 13.34 |
| IFI120 | 800 | no | - | 0.1 | - | + | + |  | 25.5 |
| IFI121 | 600 | no | - | 0.1 | - | + | + |  | 36.39 |
| H038 | 1000 | not classifiable | *A. fumigatus* | 0.54 | - | + | - | *Candida inconspicua*  *Sacchromyces cerevisae* | 23.54 |
| H043 | 1000 | not classifiable | - | 2.95 | - | + | - |  | 22.78 |
| H045 | 1000 | not classifiable | - | 1.61 | - | + | - |  | 23.63 |
| H065 | 1000 | not classifiable | - | 7.19 | - | + | - |  | 29.28 |
| H068 | 1000 | not classifiable | *A. fumigatus* | 3.11 | - | + | - |  | 23.47 |
| H069 | 1000 | not classifiable | - | 7.05 | - | + | - |  | 30.03 |
| H072 | 1000 | not classifiable | - | 3.09 | - | + | - |  | 25.02 |
| H073 | 1000 | not classifiable | - | 4.38 | - | + | + |  | 22.61 |
| H076 | 1000 | not classifiable | - | 5.54 | - | + | - |  | 25.09 |
| H079 | 1000 | not classifiable | - | 4.66 | - | + | - |  | 29.85 |

**Supplementary Table 4.** Full Patient sample breakdown vs U-dHRM results.


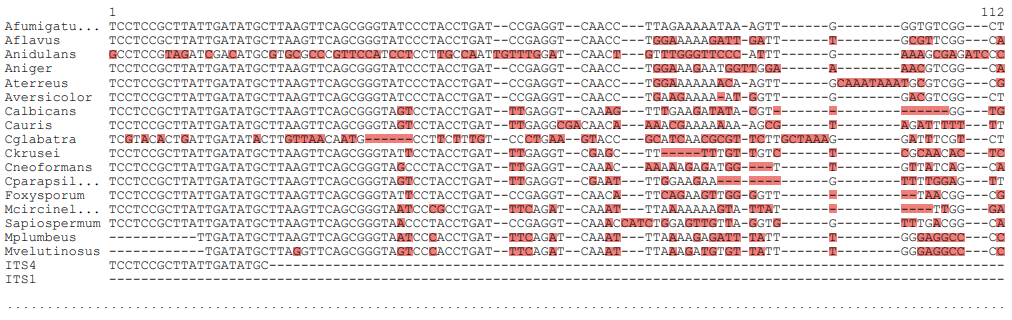


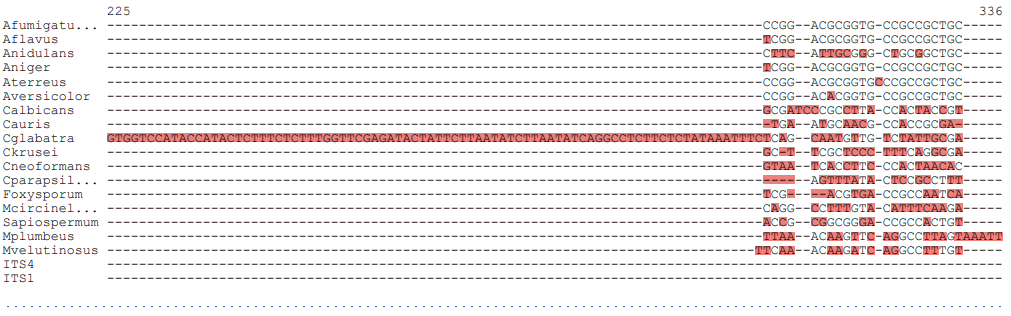


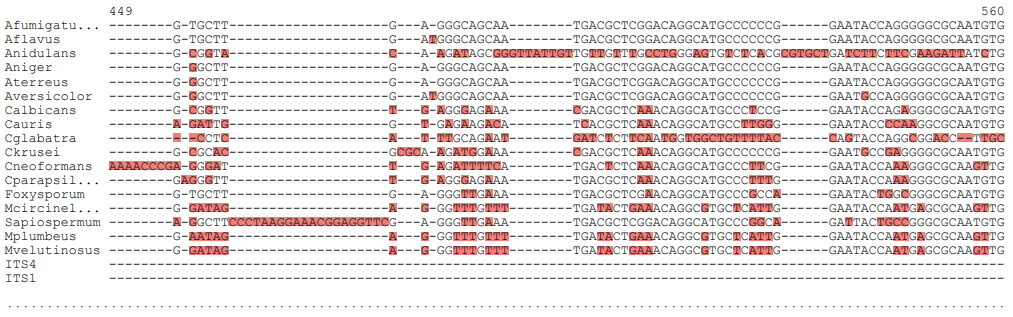


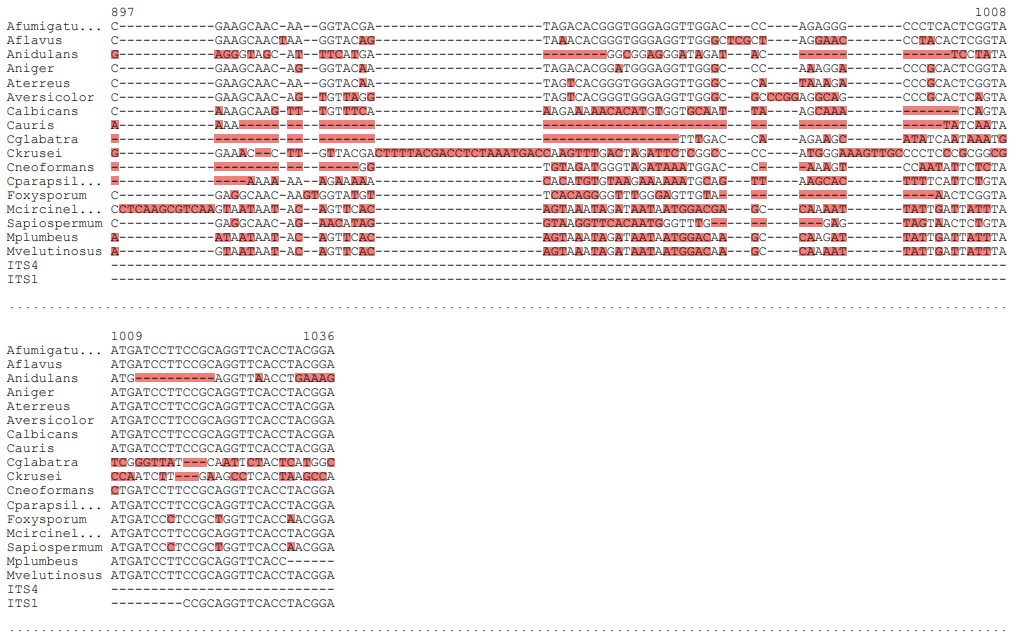


**Supplementary Figure 1.** Alignment of ITS primers to 17 clinically relevant fungal organisms.


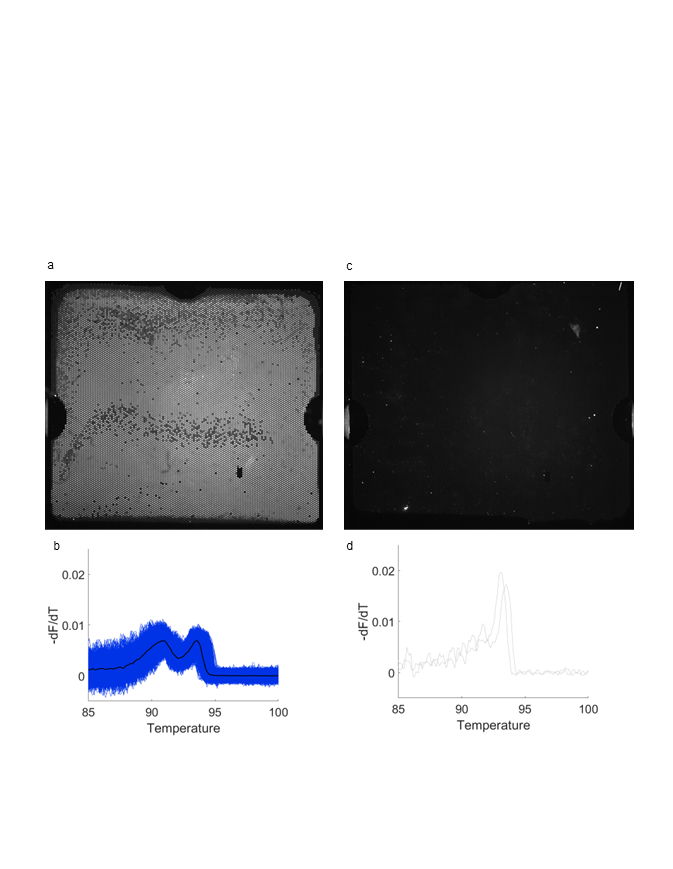
**Supplementary Figure 2. U-dHRM ITS primers only amplification.** a,b) Fluorescent micrograph of chip and melt curves produced from amplifying *C. albicans* DNA respectively. c,d) Fluorescent micrograph of chip and melt curves depicting unsuccessful *A. fumigatus* amplification using ITS primers only.


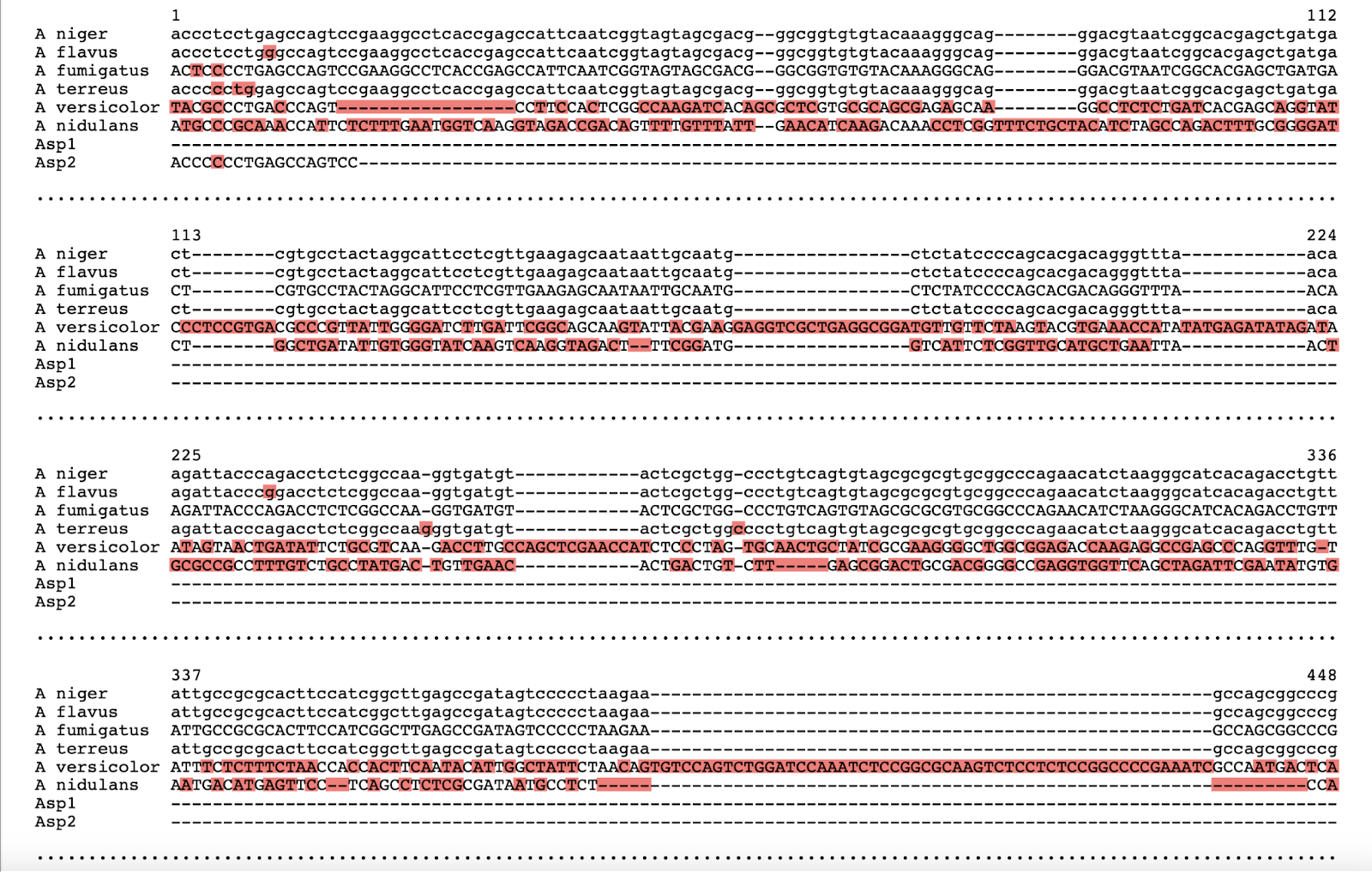


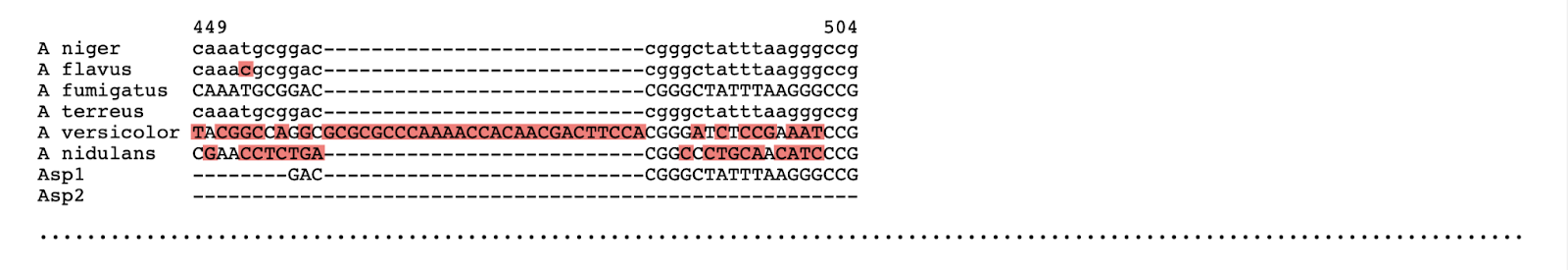


**Supplementary Figure 3.** Alignment of *Aspergillus* specific primers to 6 *Aspergillus spp.*


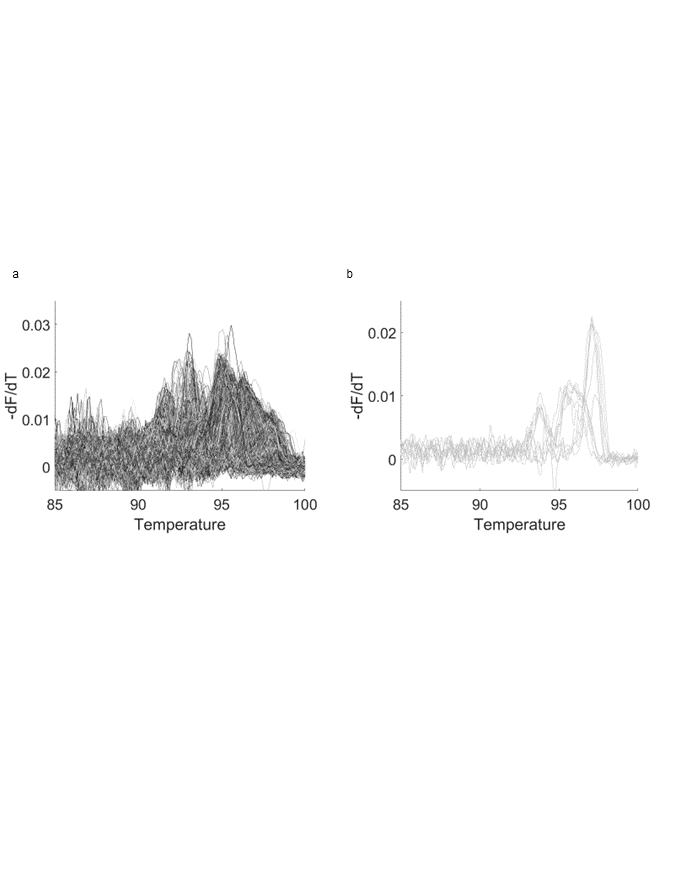


**Supplementary Figure 4. Excluded organisms.** a) *S. apiospermum* isolates demonstrated high variability in digital melt curves suggesting multiple organisms. b) *Scopulariopsis* organisms demonstrated no/low amplification, or good agreement between copy number expected vs. counted.


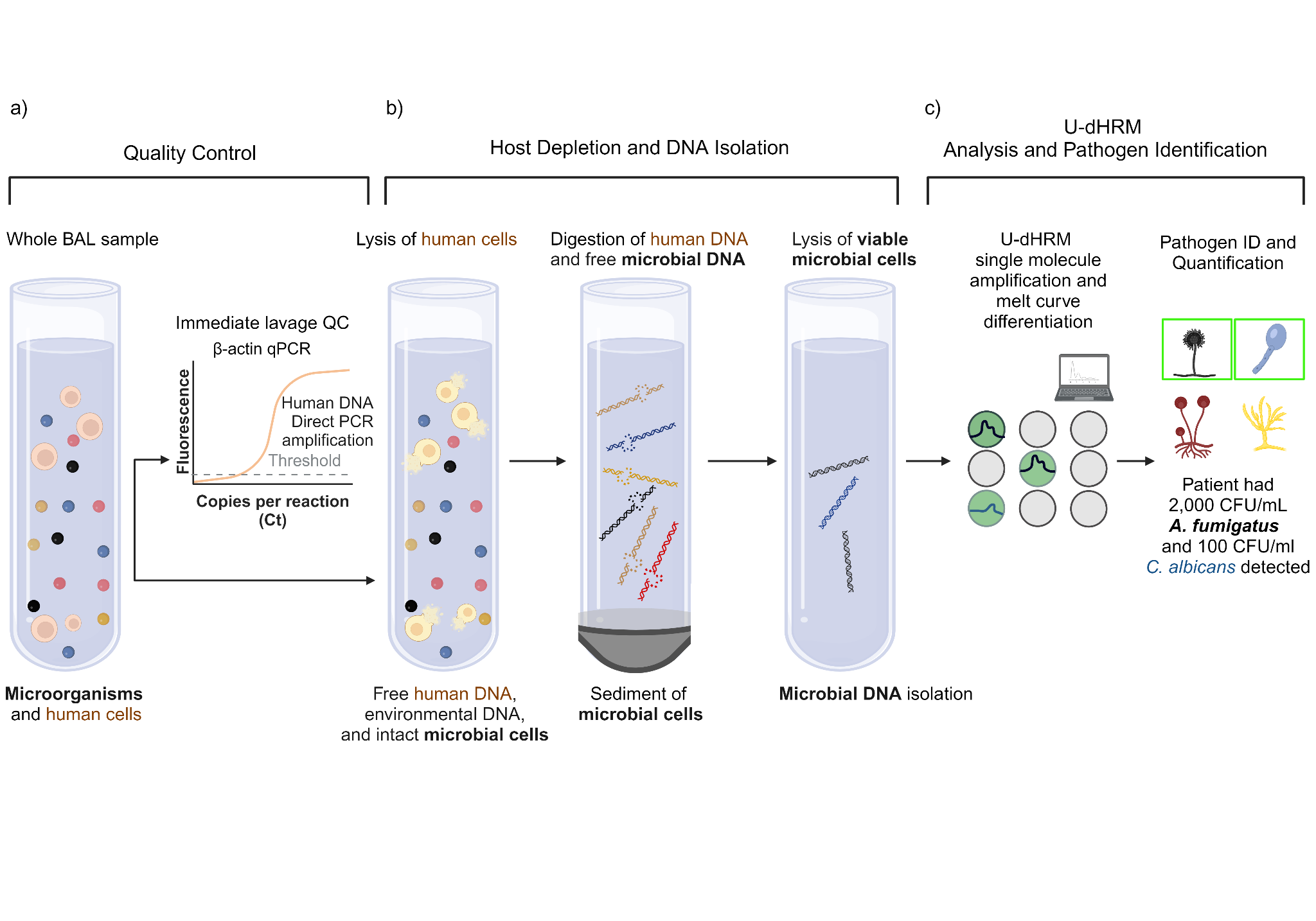


**Supplementary Figure 5. Fungal U-dHRM Process Flow.** a) Before extraction human DNA is amplified by direct PCR to assess lavage quality. b) Human cells are selectively lysed and cell-free DNA is degraded, leaving viable microbial DNA. c) U-dHRM pipeline analysis of amplified microbial DNA. Created with BioRender.com


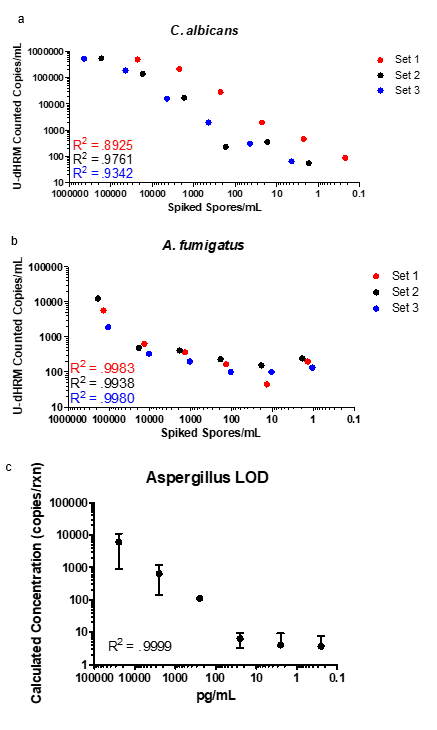


**Supplementary Figure 6. Analytical Validation Testing.** a) BALF 1x10^5^-1x10^0^ CFU/mL and no spike control spike-in dilution series of C. albicans b) BALF spike-in dilution series of *A. fumigatus* c) 2.5x10^4^-2.5x10^-1^ pg/mL DNA dilution series of *A. fumigatus* by picogreen. Plots and analysis made with GraphPad Software, Boston, Massachusetts USA, www.graphpad.com


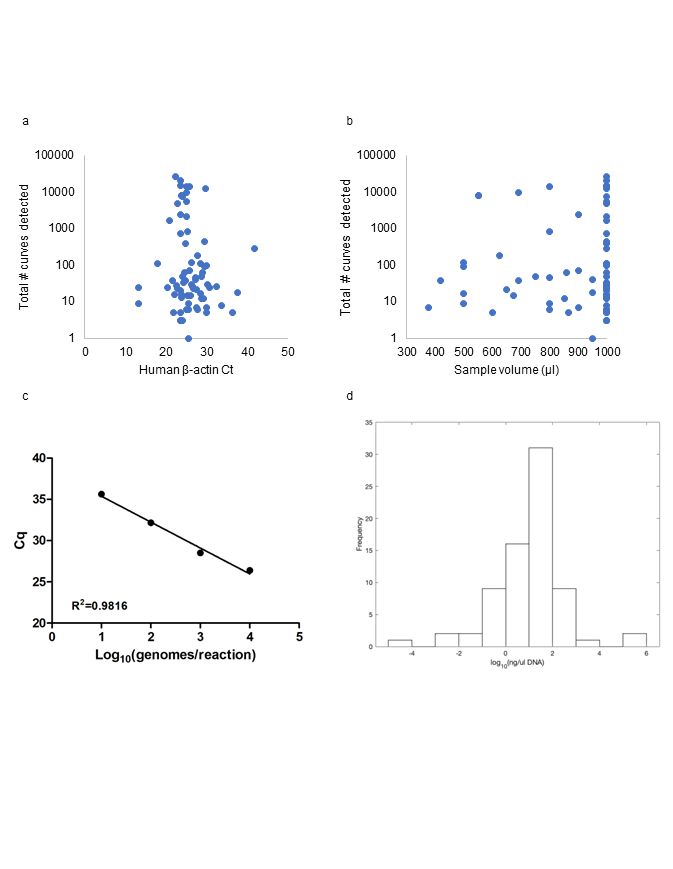


**Supplementary Figure 7.** a) Comparison of human β-actin Ct and concentration of fungi detected. b) Comparison of sample volume and concentration of fungi. c) analytical dilution series of human DNA and β-actin detection d) Distribution of human DNA observed in clinical samples


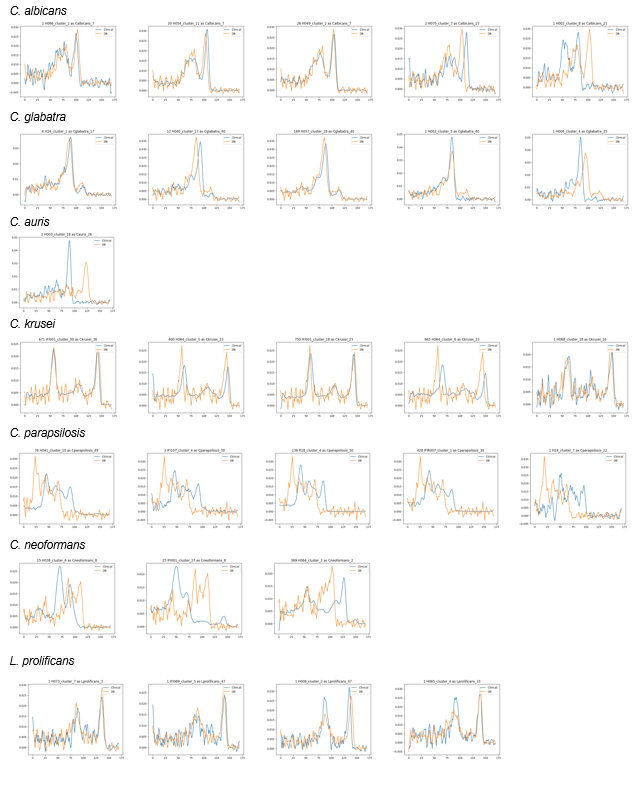


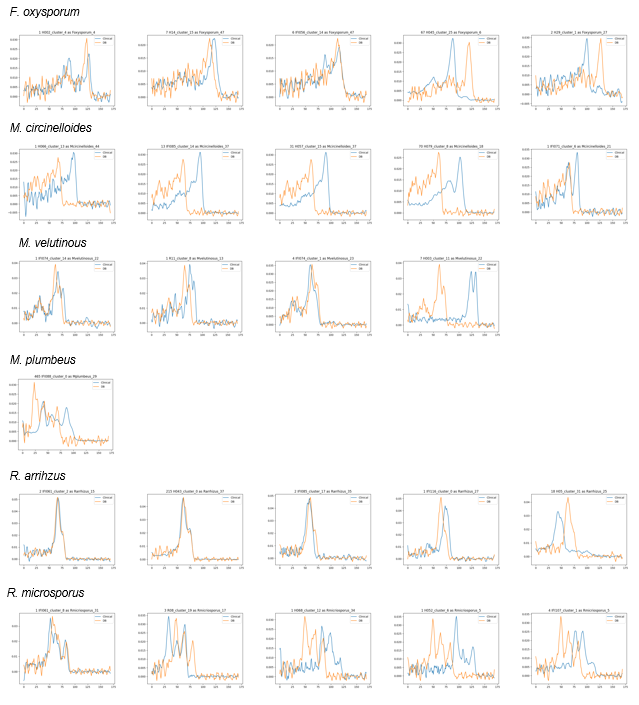


**Supplementary Figure 8**: Examples of averaged clinical curve clusters matching representative database averaged clusters.


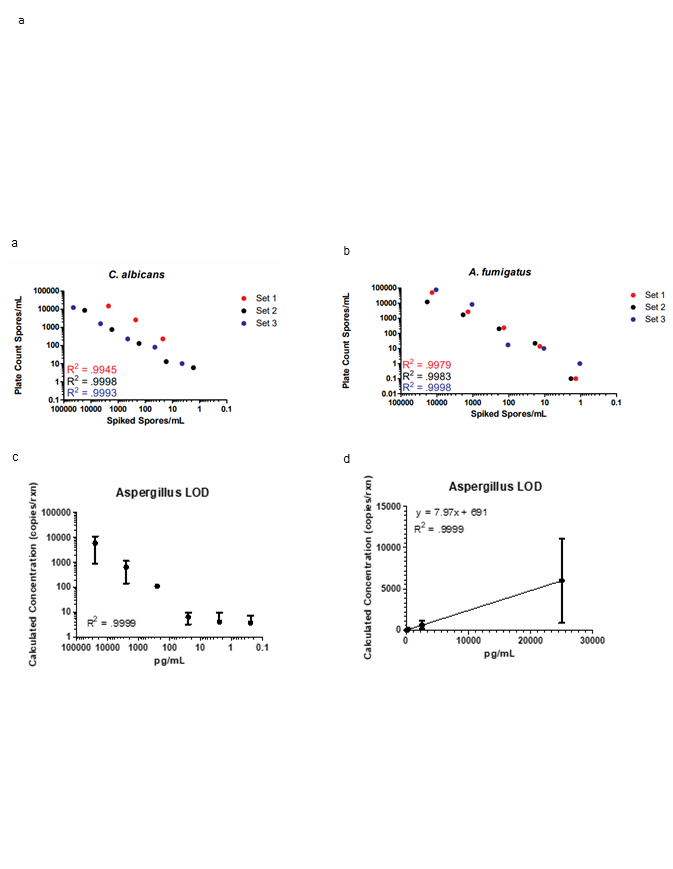
**Supplementary Figure. 9 Analytical Validation Testing**: Plate counts vs spike in, Aspergillus picogreen dilution

a) BALF plate counts vs. 1x10^5^-1x10^0^ CFU/mL and no spike control spike-in dilution series of C. albicans b) BALF spike-in dilution series of *A. fumigatus* c) 2.5x10^4^-2.5x10^-1^ pg/mL DNA dilution series of *A. fumigatus* by picogreen. Plots and analysis made with GraphPad Software, Boston, Massachusetts USA, www.graphpad.com
